# Spatial and temporal variation in surface nitrate and phosphate in the Northern Gulf of Mexico over 35 years

**DOI:** 10.1038/s41598-024-58044-4

**Published:** 2024-03-27

**Authors:** Kailani G. Acosta, Andrew R. Juhl, Ajit Subramaniam, Solange Duhamel

**Affiliations:** 1 Lamont-Doherty Earth Observatory, Columbia University, 61 Route 9W, Palisades, NY 10964 USA; 2 Department of Molecular and Cellular Biology, University of Arizona, Tucson, AZ 85721 USA

**Keywords:** Marine biology, Element cycles

## Abstract

Dissolved inorganic nutrient concentrations in the surface waters (0 to 5 m) of the Northern Gulf of Mexico (NGoM) were analyzed from 1985 to 2019 (> 10,000 observations) to determine spatiotemporal trends and their connection to nutrients supplied from the Mississippi/Atchafalaya River (MAR). In the NGoM, annual mean dissolved inorganic P (DIP) concentrations increased significantly over time, while dissolved inorganic N (DIN) concentrations showed no temporal trend. With greater salinity, mean DIN:DIP decreased from above the Redfield ratio of 16 to below it, reflecting DIN losses and the more conservative behavior of DIP with salinity. Over the same time period, annual mean P (total dissolved P, DIP, dissolved organic P) loading from the MAR to the NGoM significantly increased, annual mean DIN and total dissolved N loading showed no temporal trend, and dissolved organic N loading significantly decreased. Though DIP increased in the MAR, MAR DIP alone was insufficient to explain the surface distribution of DIP with salinity. Therefore, increases in surface DIP in the NGoM are not simply a reflection of increasing MAR DIP, pointing to temporal changes in other DIP sources. The increase in NGoM DIP suggests greater N limitation for phytoplankton, with implications for N fixation and nutrient management.

## Introduction

Marine primary production is often mediated and limited by the bioavailability of dissolved nutrients such as nitrogen (N) and phosphorus (P)^1–3^. Studies have shown that N limitation of marine primary production is more widespread than P limitation^3,4^, though P availability may play an important role over long time scales^5,6^, and in certain locations, such as the Northern Gulf of Mexico^7^. Surface ocean N and P concentrations are spatially and temporally variable as a result of many complex processes such as uptake by phytoplankton and bacteria, including luxury consumption^8^, N fixation^9^, N loss through denitrification in low oxygen regions^10^, biological and chemical conversion of inorganic and organic N and P^11,12^, legacy nutrients stored in the landscape^13,14^, and external anthropogenic inputs^15,16^. These processes can lead to deviations in organic matter production and dissolved nutrient ratios from the canonical 106C:16N:1P of Redfield proportions^3,12,17^. For example, rivers, estuaries, and coastal regions typically have higher dissolved inorganic N and P (DIN and DIP) concentrations and DIN:DIP than offshore regions^18^, where average surface DIN:DIP is about 13^5^. Meanwhile, in the majority (~ 78%) of the world’s large rivers, DIN:DIP exceeds 16, and increases with DIN concentrations^19^. These spatial patterns could be explained by net relative losses of DIN^5,20^, and/or by a relative net gain of DIP from bioconversion of dissolved organic P (DOP) to DIP as salinity increases from nearshore to offshore waters^21,22^.

In this study, we focus on the spatial and temporal variability of surface DIN and DIP concentrations over the last 35 years in the Northern Gulf of Mexico (NGoM) along the salinity gradient from the Mississippi and Atchafalaya Rivers (MAR) to offshore oceanic waters. Annual mean MAR discharge is more than 15 times that of all other rivers that drain into the NGoM^23^, thus we focus on annual mean MAR discharge as the main source of nutrients and freshwater into the NGoM system. The size, direction, and location of the NGoM freshwater plume change in concert with varying volume and timing of river discharge as well as wind speed and direction, therefore further influencing nutrient and salinity patterns^24–26^. Riverine freshwater plumes generally extend westward in the NGoM through the Louisiana Coastal Current, though wind forcing pushes buoyant plumes eastward depending on the time of year^23,27^. Physical drivers such as onshore winds and salinity cause MAR plume waters and its nutrients, sediments, and organic matter to be transported westward alongshore and eastward along the approximately 200 m depth shelf break^27,28^. The majority of riverine N and P are retained in nearshore regions of the NGoM in the fall and winter^29^, and spread offshore in the summer, though N typically declines more dramatically as a function of salinity than P^30,31^. When averaged annually, the majority of surface water DIN (70%) is retained on the shelf, while 30% of DIN is transported further offshore^32^. Together the MAR are the main sources of freshwater and nutrients into the NGoM, on average delivering 80% of the freshwater, 91% of the N loading, and 88% of the P loading into the system with a combined mean flow of approximately 21,500 m^3^ s^−1^^33–35^.

Over the last 200 years, many aspects of the MAR watershed have been altered by changing water demands, fluctuating sediment yields, navigational amendments, and flood-control systems^36^. The MAR’s water quality and chemistry has been substantially impacted by changes in land use, agriculture, industry, and sewage effluent^37,38^. From the 1950s to 1990s, TDN loading (primarily driven by increasing DIN loading) from the MAR to the NGoM tripled, and TDP loading doubled^35,36^. Since then, TDN loading has not appreciably increased, and has even stabilized in some locations^35,39^. Earlier studies found no temporal trends in DIP or TDP from the 1970s to 1990s^40^. Temporal trends in MAR nutrient loading are similar to global trends in the latter part of the twentieth century, though MAR N fluxes increased more, and P fluxes increased less, than the global average^41^.

Despite the increase in N loading from the MAR, empirical studies indicate a predominance of N limitation of phytoplankton in the NGoM^11^, and isotopic evidence indicates that the majority of N incorporated into planktonic biomass in the NGoM originates from MAR loading^35^. Nevertheless, observations of P limitation have been reported, especially at intermediate salinities within the MAR plume during spring and summer^42–44^. Multiple studies have investigated the connections between MAR flow and NGoM nutrient concentrations^31,45–47^. Lohrenz et al. (1999) found a positive correlation between MAR river discharge and MAR N:P, and in their 1990 study concluded that riverine nutrient supply constraints were a controlling factor of biomass and production at high salinities. Wysocki et al. (2006) further established that the spatial distribution of NGoM nutrients changed with MAR flow, with higher NGoM nutrient concentrations observed further offshore during periods of higher discharge. However, Cardona et al. (2016) concluded that MAR discharge alone was insufficient to predict NGoM surface nutrient concentrations, given low nutrient concentrations observed following high flow periods.

Additionally, MAR discharge and nutrient flux are tied to the spatial and temporal variability of the summer hypoxic area, or “dead zone” in the NGoM (characterized by dissolved oxygen content of < 2 mg L^−1^)^48^. The increase in MAR DIN loading from the 1950s to 1990s coincided with increased NGoM primary production, sediment C accumulation, and hypoxia—hypoxia did not appear as widespread or recurrent prior to the 1950s increase in DIN^35,49,50^. The areal extent of the NGoM dead zone is also correlated with MAR DIN loading and with primary production only in the MAR plume, not the full shelf area of the GoM^35,44,51^. Over time, the relationship between DIN loading and hypoxic area has changed, with the same amount of DIN loading in recent years leading to larger hypoxic areas than prior to the early 1990s^52,53^. Despite the importance of DIN in NGoM hypoxia, model simulations show that P limitation may play an important role by shifting primary production downstream within the plume and decreasing the area of hypoxic bottom water, due to changes in where primary production occurs and whether respiration occurs in the sediment or water column^54^. Field studies are consistent with model findings, as P limitation of phytoplankton delays the assimilation of riverine DIN in the summer and drives primary production over a larger region beyond shelf plume waters^44^.

The purpose of this study was to understand the patterns of DIN, DIP, and DIN:DIP on multiple scales in the NGoM; temporally (1985 to 2019) and spatially (shelf to offshore) in surface waters (0 to 5 m). This study represents an expanded view of nutrient trends and spatial patterns in the NGoM in the context of ongoing efforts to manage MAR nutrient loads to the NGoM, especially for N^55^. Many other studies have characterized the surface nutrient trends in the NGoM, though more have focused on more limited areas or timespans^31,36,56^. Based on our temporal and spatial nutrient analyses, we address the following objectives: (a) to delineate spatiotemporal trends in surface nutrient (DIN and DIP) concentrations and in the resulting DIN:DIP in the MAR and NGoM from 1985 to 2019; (b) to evaluate MAR nutrients as potential drivers of NGoM nutrient change over time and space; and (c) to determine whether nutrient shifts described in previous studies have persisted (i.e., changes in nutrient regime, anthropogenic impacts). These objectives aim to examine how changes in nutrient delivery through the MAR contribute to variations in surface nutrients in the NGoM over time and space.

## Methodology

### Data description

**Figure 1:**
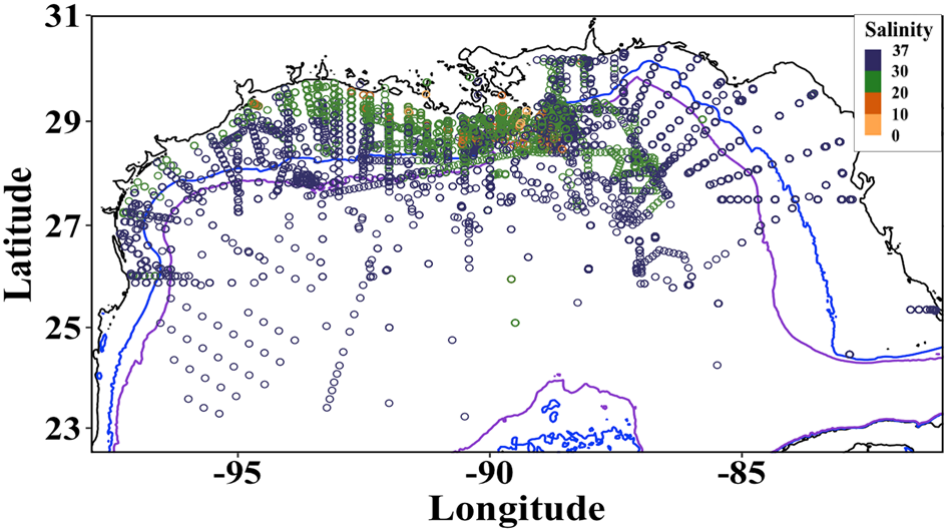
NGoM nutrient sampling locations 1985–2019. Circles represent individual surface samples (0–5 m collection depth). Circle color denotes sample salinity. Bathymetry isobaths denote the 60 m (blue line) and 200 m (purple line) depth isobaths, respectively.

The NGoM nutrient data set was compiled following methods used in Cardona et al. (2016), and enhanced by including a larger salinity range (i.e., including salinities lower than 11 ppt), adding additional nutrient samples post-2012, and other pre-2012 observations that had not been included in the earlier study. Our study’s data set included only surface (0 to 5 m collection depth) nutrient data in the NGoM (defined as coordinates − 98˚, − 79˚ to 22.5˚, 31˚; Fig. 1) from 1985 to 2019, with most data collected in the summer months (Fig. S2). NGoM surface data were used to ensure that samples were influenced by the MAR plume. Numerous studies of nutrients, phytoplankton, and riverine transport have entirely or primarily focused on the upper few meters of the NGoM water column (e.g., Cardona et al., 2016, Wysocki et al., 2006). Thus, the results of this study elaborate on the context provided by earlier work.

The criteria for the compiled NGoM surface nutrient data included: 0 to 5 m sampling depth, collection date from 1985 to 2019, and study location in the northern portion of the GoM (within coordinate box listed above). Data also needed to include salinity (0–37 ppt), and surface DIN and DIP concentrations in µM, if data were not listed in µmoles/L or µM, they were converted to µM. For quality assurance, data with salinities greater than 37 ppt or without corresponding salinity values were excluded (i.e., this excluded all data points from cruises from 1988 and 2014). In addition, 39 NGoM datapoints (from n = 10,007 total) with nutrient values above 8 µM DIP or 110 µM DIN were excluded as they were greater than three standard deviations above the mean. The bottom depth for each sample coordinate was calculated with the *marmap* R package for all data within coordinates − 98°, − 79°, and 20°, 31°^57^. These data were compiled from a variety of sources with varied collection and analysis data validation methods; with this in mind, we verified the data in comparison to data from other years and regions, covering a large portion of the surface waters of the NGoM over 35 years. Data with these criteria were compiled from the Biological & Chemical Oceanography Data Management Office (BCO-DMO), National Oceanic and Atmospheric Administration National Centers for Environmental Information—World Ocean Database (NOAA NCEI WOD), United States Geological Survey (USGS), and Gulf of Mexico Research Initiative Information and Data Cooperative (GRIIDC) (Table S1).

Uptake rates of NH_4_^+^ in the MAR plume can be comparable to those for NO_3_^-^; however, we operationally defined DIN as the NO_2_^−^ + NO_3_^−^ concentration, excluding NH_4_^+^ because it was relatively rarely measured in the MAR and NGoM databases and was often near or below the detection limit or a minor fraction of DIN when it was quantified. Typically, NH_4_^+^ is rapidly transformed into NO_2_^−^ + NO_3_^−^, with concentrations from 0.17 to 0.44 µM and little spatial variability in both the lower MAR and NGoM^58,59^. Prior studies of this region similarly focused on NO_2_^−^ + NO_3_^−^. USGS historical records for the Mississippi River at St. Francisville, LA, that we used in our MAR nutrient comparison^31,60,61^, also define inorganic N as NO_2_^−^ + NO_3_^−^. The majority of surveyed studies did not measure organic forms of N and P, so analyses were conducted only using inorganic nutrient data.

For some analyses, the surface nutrient data were subset into three different spatial regions: hypoxic region (data within − 88° to − 95° and 27° to 29° with bottom depths of < 60 m, Figure S3a), shelf region (data with bottom depths of < 200 m, Fig. S3b), and offshore region (data with bottom depths of > 200 m, Fig. S3c). The hypoxic region defined here was a shallow subset of the shelf region where hypoxic bottom waters are most likely to be found; the boundaries of the hypoxic region did not change through time in these analyses, though the actual area measured with hypoxic bottom water varies seasonally and annually^35^.

MAR data (nutrient loading, nutrient concentrations, discharge, and suspended sediment concentrations) were also compiled for 1985 to 2019 from the St. Francisville, Louisiana USGS National Water Quality Network (NWQN) program monitoring station because it had the longest running nutrient data (dissolved nutrients measured multiple times per year since 1954) in the lower portion of the MAR, and it is located close to the point where the Atchafalaya River diverges from the Mississippi, covering a drainage area greater than 2.9 × 10^6^ km^2^^62^. Many other NGoM studies similarly use MAR nutrient data from the St. Francisville, LA USGS monitoring station^36,63,64^. In addition to DIN (NO_2_^−^ + NO_3_^−^ as N, filtered, mg L^−1^) and DIP (orthophosphate, filtered, mg L^−1^), the USGS MAR water quality data included TDN (NO_2_^−^ + NO_3_^−^ + NH_4_^+^  + organic-N, filtered, mg L^−1^), TDP (filtered, mg L^−1^), dissolved organic N (DON; filtered, mg L^−1^), and dissolved organic P (DOP; filtered, mg L^−1^), that were not included within the NGoM nutrient data set because they were rarely measured compared to DIN and DIP in the NGoM. MAR data were compiled for nutrients (in mg L^−1^), discharge (in tons, then converted to kg year^−1^), loading (in tons, then converted to kg year^−1^) and suspended sediments from all available dates between 1985 and 2019.

The MAR basin has been a location of intense streamflow and large-scale water quality monitoring for decades; this study tests whether the MAR nutrient fluxes can explain spatial and temporal nutrient trends in the NGoM as a whole. MAR St. Francisville water quality loads are sourced from USGS (USGS Station 07373420) as indicated in Lee (2022). USGS computed annual, flow-normalized water-quality loads (mean annual sample n = 16) using the USGS Load Estimator (LOADEST) program and Weighted Regressions on Time, Discharge, and Season method (WRTDS) between 1985 and 2019 using available discrete water-quality and streamflow information^61^. Nutrient fluxes were calculated using Adjusted Maximum Likelihood Estimation (AMLE) using the LOADEST program to compute nutrient loads using a 10-year moving window approach in the MAR basin^61,65^. Load estimation methods included the log of cubic streamflow, time (annual, seasonal, monthly), and historical streamflow conditions^61^. The WRTDS water quality data for the MAR is used by the Mississippi River/Gulf of Mexico Hypoxia Task Force to meet their goals of reducing the hypoxic zone in the GoM to a 5 year moving average of 5000 km^2^^61,65^. To determine the annual amount of MAR discharge at St. Francisville, annual flow for a given water year was converted from daily cubic meters second^−1^ to acre-feet day^−1^ then averaged annually. For WRTDS loads calculated for the MAR at St, Francisville, WRTDS calibration records existed from 1980 to 2019 for loads of TDN, TDP, DIN, and suspended sediment concentrations, while DIP loads were analyzed and calibrated from 1982 to 2019^61,65^. These WRTDS loads are assumed to be the most accurate load estimates for any given year^65^. In our study, the MAR annual WRTDS nutrient load data was compared to MAR nutrient data using comparable analyses to determine correlations and compare trends in nutrient concentrations over time and space.

### Data analyses

All statistical analyses used a significance level of 0.05. NGoM temporal nutrient trends were evaluated by linear regressions of annual mean DIN and DIP concentrations and DIN:DIP against time. Similarly, temporal trends in MAR data were assessed using linear regressions of annual means for each parameter over time (nutrient loading, nutrient concentrations, discharge, suspended sediment). Temporal regression analyses in this study set 1985 as year zero so that the regression equations provided meaningful y-intercepts. Oftentimes, long successive time-series contain autocorrelation of data^66^. To remove potential autocorrelation in the time series data, we based the analysis on annual mean NGoM and MAR nutrient concentrations, and also tested for autocorrelation using Durbin-Watson tests. For data with significant autocorrelation, Cochrane-Orcutt transformation was used, and Durbin-Watson tests were run again to confirm reduction of autocorrelation below significance. The Cochrane-Orcutt estimation and subsequent transformation also accounted for heteroscedasticity in the data, confirmed by Breusch-Pagan tests. In addition to linear regression, changepoint analyses of mean annual nutrient concentrations were conducted using a regression model in R package *changepoint* with segmented relationships for annual mean DIN and DIP over time to determine whether monotonic analyses were appropriate for the nutrient time series. Changepoint analyses identify statistically significant changes or breaks in trends over time. After standardizing year and nutrient concentration (DIN, DIP) variables, we then used a Markov Chain Monte Carlo simulation to fit a Bayesian changepoint model. The changepoint in the data represents a gap or change in the distribution of the nutrient data in a given year. If there are no significant changepoints, then monotonic analyses are best suited for the data.

Pearson correlation analyses tested for significant temporal relationships between annual mean MAR discharge, nutrient concentrations, and nutrient loading with the corresponding annual mean NGoM nutrient concentrations. Parallel analyses focused on each of the three regional data subsets: hypoxic, shelf, and offshore regions. Pearson correlation analyses also compared annual mean nutrient concentrations in the shelf and offshore regions to each other. Finally, Pearson correlation analyses compared the annual area of hypoxic bottom water^35^ to mean annual MAR nutrient loading and mean annual NGoM nutrient concentrations as a whole, as well as in each spatial region.

Next, the NGoM nutrient data set was analyzed in relation to sample salinity to incorporate dilution along the continuum from the MAR endmember to the oceanic endmember into the analyses of nutrient concentrations. While there was generally a trend of increasing salinity going offshore, it should be noted that nearly the full range of salinities was found in both shelf and offshore regions, and salinity plots were thus not strictly analogous to geographic patterns (i.e., some samples found beyond the 200 m isobath had salinities less than 20; Fig. S2). Low salinity offshore waters were located within the freshwater plume, which varies over time and space with environmental variables such as river discharge, wind speed, and direction^67^. Nutrient data were regressed against salinity using a variety of functions, including linear, exponential, and power functions; within monotonic functions, bivariate linear regression of ln-transformed data provided the highest r^2^ values and significance. To account for zero values in the data set prior to the ln-transformation, we added a reasonable detection limit to all samples (0.05 µM for DIN, 0.03 µM for DIP), similar to that used in Cardona et al. (2016). Linear regressions of ln[DIN], ln[DIP], and ln(DIN:DIP) vs. salinity described the general trends of decreasing nutrient concentrations along the salinity gradient due to dilution and loss (e.g., phytoplankton uptake). To improve visualization, especially of low nutrient concentrations, figures show untransformed data plotted with log-scale y-axes. These regressions and their presentation on plots were primarily intended to show general trends of nutrient concentrations with salinity.

Nutrient data were also compared to conservative mixing functions, calculated by linearly connecting the long-term mean MAR TDN, TDP, DIN, and DIP concentrations at St. Francisville, LA at 0 ppt salinity to the minimum ocean endmember nutrient concentration (the detection limits mentioned above) at 37 ppt (Fig. S1). This line described the expected decline of nutrient concentration due to dilution of MAR water with the offshore endmember. Note that on the semi-log plots shown in later figures, the mixing functions appear curved. To understand how the relationship between nutrient concentrations and salinity changed through time relative to the conservative mixing function, we calculated the residuals of each data point compared to the respective conservative mixing function along the salinity gradient. Negative residuals signified loss of nutrients relative to the MAR endmember that exceeded the decline expected due to mixing with the offshore endmember (e.g., due to phytoplankton uptake). Positive residuals indicated excess inorganic nutrients relative to the two-endmember function, suggesting a source in addition to the MAR endmember. Analogous residual analyses were also conducted on the hypoxic, shelf, and offshore regional subsets.

## Results

### Spatiotemporal changes in annual mean NGoM DIN, DIP, and DIN:DIP

**Figure 2:**
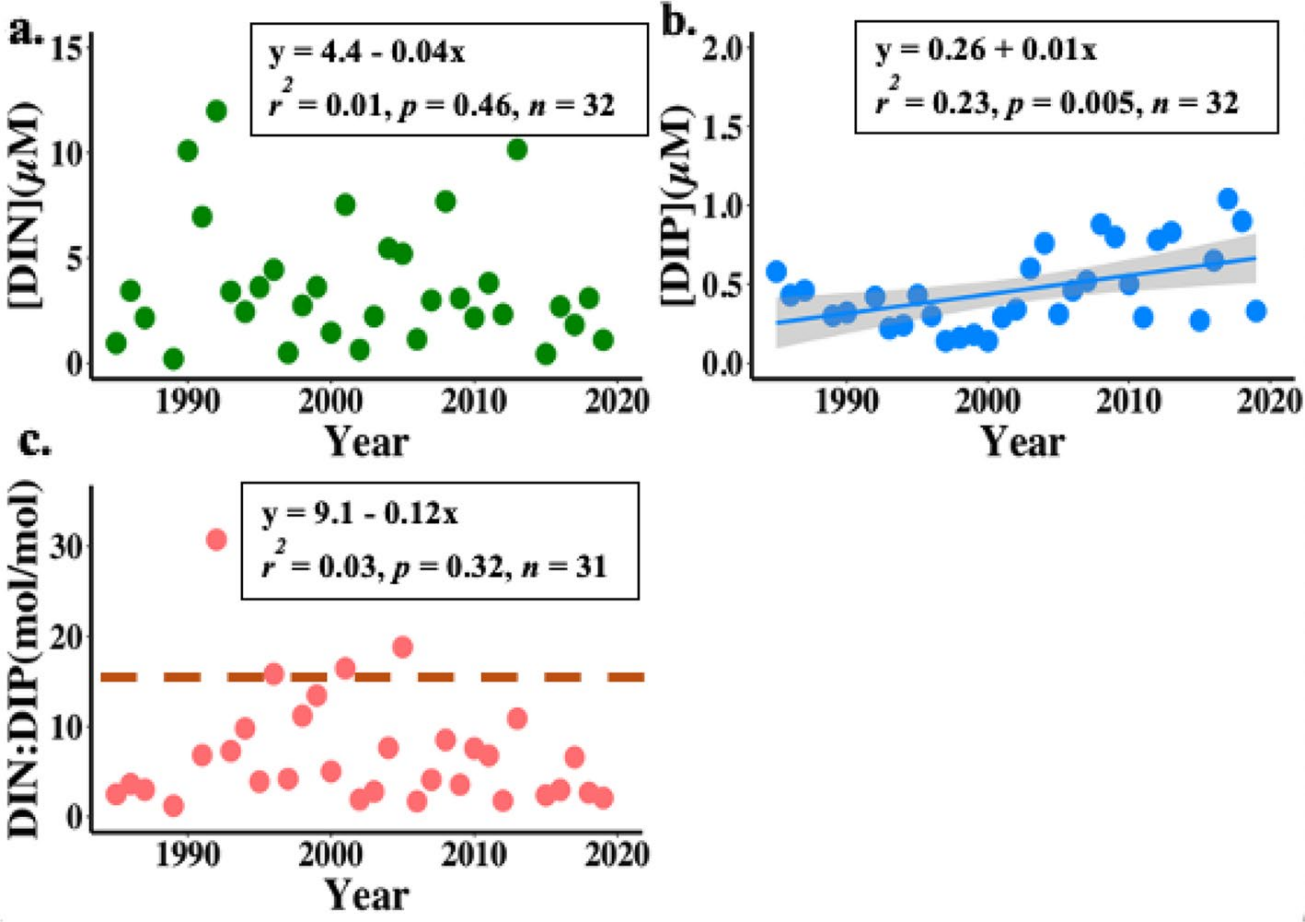
Annual mean NGoM nutrient concentrations 1985 – 2019; (a) DIN (green), (b) DIP (blue), and (c) DIN:DIP (molar ratio, pink). The DIP linear regression was significant and is shown with shaded gray 95% confidence interval. The Redfield ratio of DIN:DIP = 16 is highlighted by a dashed orange line in (c).

NGoM DIN concentrations (1985–2019) were highly variable and showed no significant temporal trend (Fig. 2a), while DIP concentrations increased significantly (Fig. 2b). DIN:DIP also showed no significant temporal trend, though DIN:DIP ratios from 2015 to 2019 were among the lowest of the study period, with a mean DIN:DIP of 3.3 (Fig. 2c). Low DIN:DIP values (from 1.2 to 3.6) also occurred in the late 1980s. Durbin-Watson autocorrelation tests of annual mean nutrient concentrations over time showed no autocorrelation for annual DIN means over time, and minimal autocorrelation for annual DIP means over time (Table S4). Thus, Cochrane-Orcutt estimations were run on the annual DIP means to transform the data and remove autocorrelation, then Durbin-Watson autocorrelation tests were run again to confirm there was no longer significant autocorrelation. The transformed DIP data had the same r^2^ value, *p*-value, and slope as the untransformed data, and therefore the untransformed data is shown in Fig. 2 for clarity. In addition, the transformation removed heteroscedasticity in the DIP means over time, which was confirmed with the Breusch-Pagan test. Bayesian changepoint analyses of DIN and DIP over time showed that there were no years when there was a significant changepoint in the time series; though there was a decrease in the changepoint time statistic for DIN at 2013 and an increase in the changepoint time statistic for DIP at 1999, the error bars for each were large and included much of the time series (Fig. S5). There were also no significant temporal DIN or DIN:DIP trends in the regional subsets; however, hypoxic and shelf DIP concentrations significantly increased over time (Fig. S6; Table S3).

### Annual mean MAR nutrient loading into the NGoM

**Figure 3:**
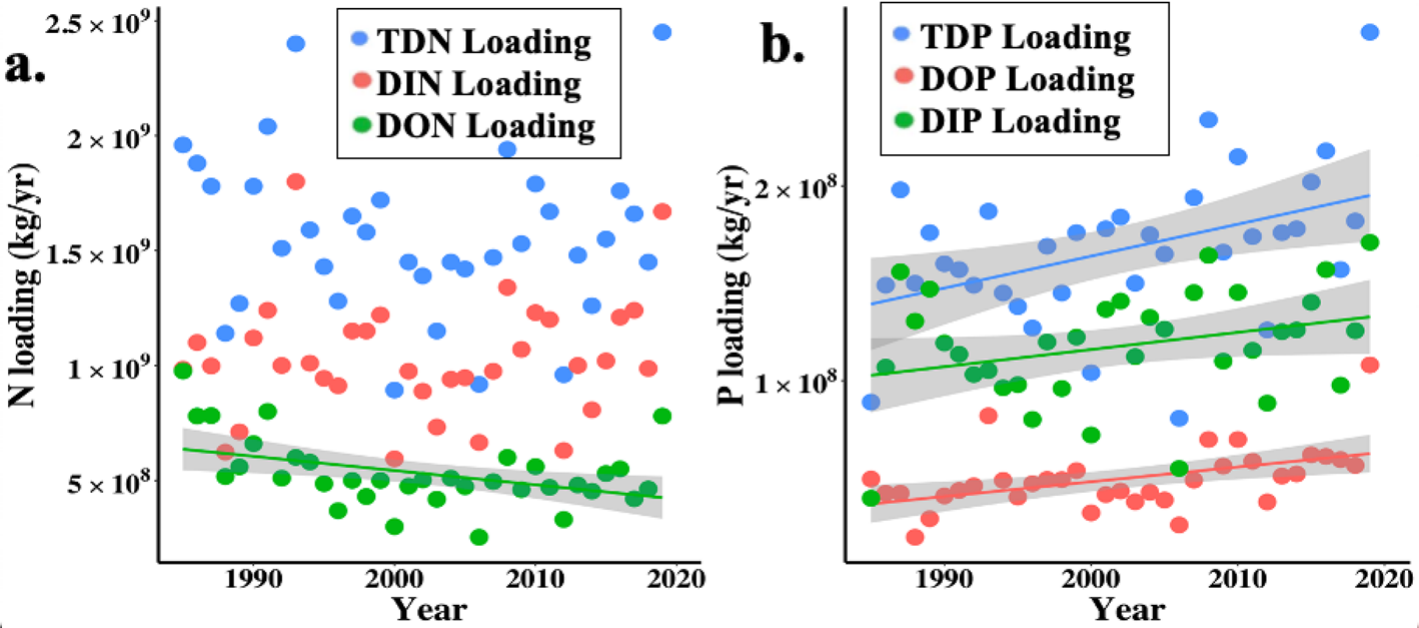
Annual mean (**a**) N loading and (**b**) P loading from the MAR into the NGoM 1985–2019. Non-significant regressions are not shown. Gray shading represents the 95% confidence interval for the statistically significant regression lines. See supplemental Table S1 for corresponding linear regression equations, *r*^*2*^, *p-*values, and *n*. Based on USGS data (Lee, 2022).

Similar to NGoM nutrient trends from 1985 to 2019, TDN and DIN loading from the MAR into the NGoM did not change significantly, while DON loading significantly decreased (Fig. 3a, Table S2). Over the same time period, TDP, DIP, and DOP loading from the MAR into the NGoM all increased significantly (Fig. 3b, Table S2). As annual mean MAR discharge did not change significantly over the study time period (Fig. S4b), it is not surprising that MAR DIN concentrations did not show any temporal trend from 1985 to 2019, although MAR DIP concentrations increased significantly (Fig. S4a). Annual mean suspended sediment concentrations, however, declined significantly during the period (Fig. S4c).

Comparing annual mean NGoM DIN and DIP concentrations to MAR discharge, MAR DIN and DIP concentrations, and MAR DIN and DIP loads produced similar patterns of significant relationships for the entire NGoM, the hypoxic region, and the shelf region (Table S4). All were significantly correlated except DIN:DIP, which did not significantly correlate to MAR discharge in the entire NGoM, hypoxic, or shelf regions.

In contrast to results for the hypoxic and shelf regions, correlation analyses for the offshore region found no significant correlations between offshore DIN and MAR discharge, MAR DIN or DIP concentrations or loading (Table S4). On the other hand, offshore DIP concentrations did significantly correlate with MAR discharge, MAR DIP concentrations, and MAR DIN:DIP. Offshore DIN:DIP was significantly correlated to MAR discharge, MAR DIN and DIP concentrations and loading, however, not to MAR DIN:DIP (Table S4). In general, shelf and offshore nutrient concentrations were not significantly correlated with each other over time, with exceptions of the significantly positively correlated offshore DIN:DIP to shelf DIN and DIP concentrations (Table S5).

The areal extent of the hypoxic bottom water in the NGoM fluctuates interannually^35^, and correlation analyses were used to determine if this hypoxic area related to nutrient observations. The annual area of hypoxic bottom water was significantly correlated with all forms of N and P loading from the MAR into the NGoM (Table S6). The annual area of hypoxic bottom water was also significantly correlated with annual mean NGoM DIN and DIP concentrations and DIN:DIP in the entire NGoM dataset, the hypoxic region, and the shelf region (Table S6). However, the area of hypoxic bottom water was not significantly correlated with offshore nutrient concentrations (Table S6).

### Nutrient versus salinity relationships in the NGoM

**Figure 4:**
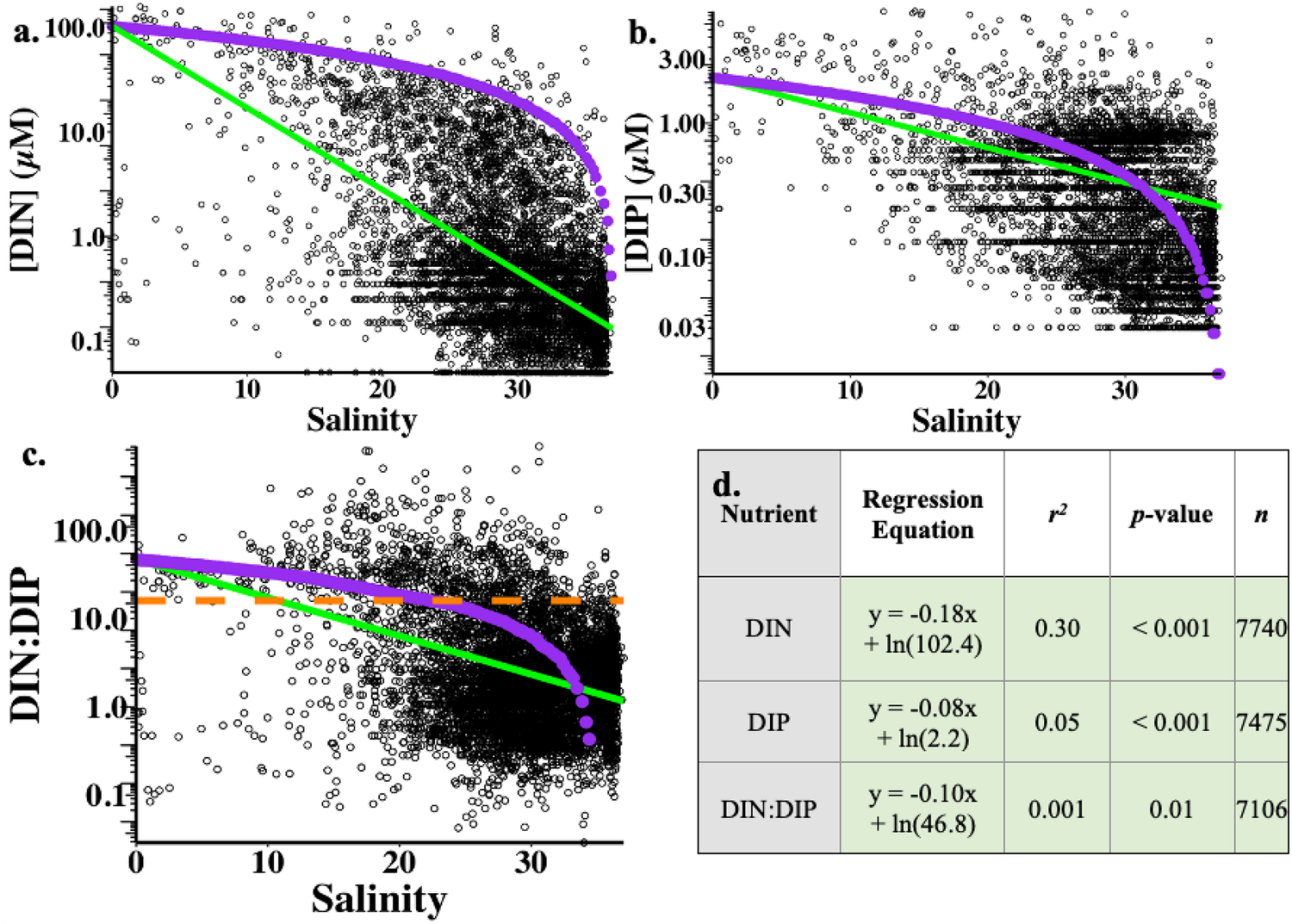
NGoM surface nutrient concentration vs. salinity 1985–2019: (a) [DIN]; (b) [DIP]; (c) DIN:DIP. Black circles (excluding 1988 and 2014) represent surface nutrient data in µM for (a) and (b), and are the molar ratio in (c). Untransformed data are shown to facilitate reading values directly from the plots, note the log scale of y-axes. The green lines represent linear regressions to ln-transformed data, with the corresponding equations, *r*^*2*^, *p*, and *n* values listed in (d). The conservative mixing functions are shown in purple. The dashed orange line in (c) indicates the Redfield ratio of 16 DIN:DIP. Light green shading in (d) highlights significant *p* values.

All NGoM DIN and DIP data (1985–2019) were plotted against corresponding sample salinity in Fig. 4 with each black circle representing a single sample. The conservative mixing function and linear best fits (to ln-transformed values) were also shown. Both DIN and DIP generally declined with increasing salinity, and the slope of the decline was steeper for DIN compared to DIP. Relative to the respective conservative mixing function (shown in purple), only 2% of the DIN values across the salinity range were above the DIN function, while about half (51.8%) of the DIP observations exceeded the DIP mixing function. DIN:DIP in the NGoM generally decreased from above to below the Redfield ratio with increasing salinity, driven by the greater decline in DIN relative to DIP concentrations (Fig. 4c). At 0 salinity in the MAR, DIN:DIP averaged 46.8, and typically declined to less than 16 within the NGoM where salinity was greater than 20.

Nutrient vs. salinity relationships were also plotted separately for the hypoxic, shelf, and offshore subsets of the data (Fig. S7; Table S6). While there were differences in the best-fit slopes for each of the three data subsets, the general patterns were similar to the overall data set. Changing the freshwater endmember across the range of DIN and DIP values from 1985 to 2019 did not affect nutrient vs. salinity relationships. For all regions, DIN declined more for a given change in salinity than DIP, and a much larger fraction of the DIP observations (relative to the DIN observations) exceeded the mixing function. Subsequent analyses subset the data shown in Fig. 4 to examine the residuals of each nutrient from the mixing functions.

**Figure 5:**
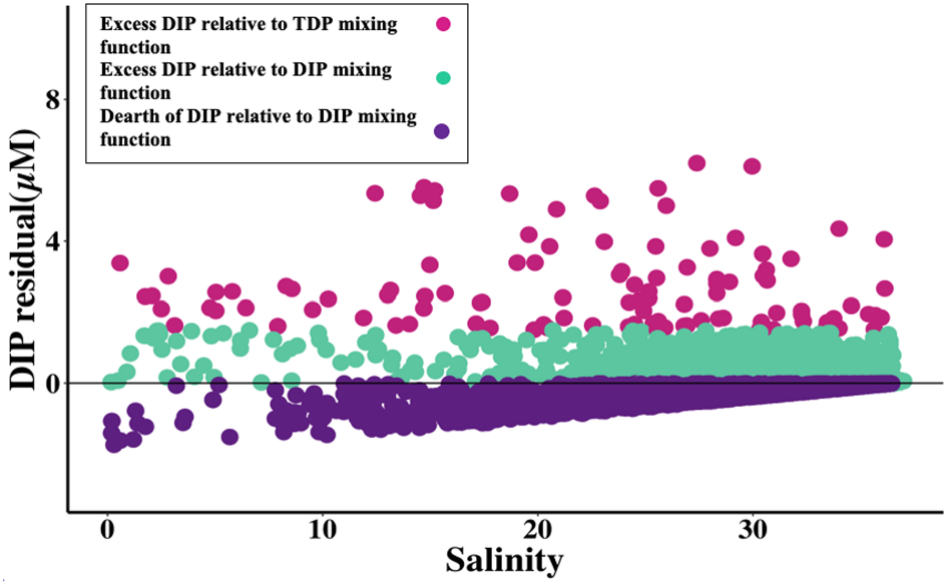
Residuals of NGoM DIP concentrations (1985–2019) relative to the conservative mixing functions connecting MAR DIP and TDP to the offshore endmember. Each point equates to an individual sample and shows the DIP residual relative to the MAR DIP mixing function (y = -0.059x + 2.2). Positive residuals (teal and pink) indicate that the actual values were higher than predicted, and negative residuals (purple) signify that the actual values were lower than predicted by the MAR DIP mixing function. Pink values are positive relative to both the MAR DIP mixing function and would also be positive relative to the MAR TDP mixing function (y = -0.089x + 3.3). Teal values are positive relative to the MAR DIP mixing function, but would be negative relative to the MAR TDP mixing function. Purple values are negative relative to both mixing functions.

Nutrient residuals relative to the conservative mixing function were calculated for each data point as the difference between actual and predicted value, with positive residuals meaning an excess or production of the nutrient while the negative residuals indicate the loss or consumption of the nutrient in addition to dilution by low-nutrient ocean water (depending on the sample salinity; Fig. 5, Figs. S8, S9). Here, we emphasize the presentation of data on the DIP residuals because over half exceeded the mixing function, in contrast to only 2% of DIN residuals. Residuals were shown relative to the DIP mixing function (y = − 0.06x + 2.2) which is zero on the y-axis. Additionally, the symbol colors reflect residual values relative to both DIP and TDP (y = −0.09x + 3.3) mixing functions, under the assumption that all TDP from the MAR could be converted to DIP in the NGoM.

## Discussion

Over time, DIP concentrations significantly increased in the NGoM, but with greater interannual variability over time. This rise in DIP was notable not only because of its increase over time, but also because DIP concentrations were frequently in excess of the MAR DIP mixing function across the salinity gradient. Even when we used annual values of MAR DIP concentrations, about half of the NGoM DIP concentrations still exceeded the mixing line. Thus, although MAR DIP loading rose over time, it was insufficient to explain the distribution of DIP concentrations in the NGoM. This contrasts to MAR DIN values, which could account for NGoM DIN concentrations along the salinity gradient because DIN concentrations were predominantly (98%) below the MAR DIN mixing function. Other studies have similarly proposed that there are excess sources of DIP (or selective removal of DIN) in the NGoM shelf region beyond the average MAR outflow area^60,68^.

A two-endmember mixing model for NGoM nutrient distributions is highly simplified and inputs from freshwater endmembers other than the MAR certainly contribute to NGoM nutrient concentrations^69^. Nevertheless, the high relative contribution of the MAR to NGoM nutrient loading^32^ and the temporal correlations between MAR nutrient loads and NGoM concentrations shown in this study support our focus on the MAR as the dominant freshwater endmember. Moreover, this two-endmember approximation does effectively highlight fundamental differences in the spatial and temporal patterns of DIN and DIP in the NGoM that require further exploration. Resolving the NGoM DIP-salinity relationship requires a process that provides DIP to the NGoM in excess of the MAR DIP source along the entire salinity gradient, that also increases through time, at least over the shelf region. In this context it is worth highlighting that the slopes of DIN, DIP, and DIN:DIP vs. salinity are nearly identical across the hypoxic, shelf, and offshore regions, suggesting that the same processes control nutrient patterns regardless of distance from shore, or bottom depth. Within the MAR DIP plume itself, relevant processes to consider include DIP regeneration and recycling, precipitation and aerosols, adsorption/desorption of P from suspended particles and benthic sediment, vertical mixing, and DOP mineralization.

DIP regeneration/recycling, the conversion of autochthonous organic P to DIP^6^, is an important source of P to NGoM phytoplankton, and P turnover times within the upper water column are rapid^70^. However, such cycling cannot by itself explain NGoM DIP concentrations that surpass the MAR DIP mixing function since regeneration of DIP from the MAR would need additional DIP sources to exceed mixing. Throughout the MAR watershed and NGoM, P is deposited from rainfall, dust, and anthropogenic emissions^71,72^. While atmospheric P deposition has increased over time in other locations^73^, the atmospheric DIP and TP contribution to the NGoM is still very small (2–10 ng m^−3^)^74^.

DIP adsorbs to particles in freshwater, and is released with increasing salinity and discharge^69,75^. However, suspended-sediment-derived P is unlikely to explain the upward trend in NGoM DIP, because the percentage of P in MAR suspended sediment stayed relatively stable over time (Fig. S4d), while MAR suspended sediment concentrations have significantly declined since 1985. P can also be remobilized into bottom waters from benthic iron or sulfate reduction, mediated by oxygen levels^76,77^. For example, during NGoM hypoxic events, TDP is released into the water column from bottom sediments^7^. Though benthic DIP sources are likely important within the MAR watershed and in shallow, nearshore areas of the NGoM^69^, our results show a pattern of excess DIP at high salinities, that are typically found over deep water columns, further offshore.

Although stratification in the NGoM can be strong^78^, the two-endmember model applied to the nutrient distributions implies continuous vertical or horizontal mixing between the endmembers. While vertical mixing transports DIP to the surface from deeper depths, it also transports DIN. To provide excess DIP to the NGoM system, the deeper waters being mixed upwards would need to have lower DIN:DIP than the offshore endmember. In addition, to align with the temporal trend in DIP, there would need to be an increase in either the mixing intensity over time, or a decrease in DIN:DIP in those deeper waters. Further research will be needed to assess these possibilities.

Biological mineralization of organic P compounds through enzymatic reactions^12,79^ or photolysis^80^ could break down organic P-containing compounds originally delivered from the MAR, adding DIP to the NGoM water column in excess of the MAR DIP mixing function. Figure 5 demonstrates that including MAR TDP inputs would be sufficient to explain most NGoM DIP concentrations if all MAR-derived DOP was converted into DIP along the salinity gradient in the NGoM. Thus, the combination of DOP and DIP loading from the MAR could account for increasing DIP concentrations in the NGoM, though other processes are not excluded. While it seems unlikely that all MAR-derived DOP would be converted to DIP, the match with the DIP data suggests that DOP mineralization is an important source of DIP along the salinity gradient. Further research quantifying the fraction and processes of MAR DOP mineralization along the salinity gradient would help constrain the degree to which MAR DOP inputs can explain NGoM DIP trends.

This study expands upon previous nutrient research in the MAR and NGoM. From 1985 to 2019, N and P loading in the MAR increased greatly relative to the 1950s^33,36^. During the period of this study, DIN loading fluctuated around a value of approximately 1 × 10^9^ kg yr^−1^, while DON loading decreased significantly by approximately 20% from 1985 to 2019. Stabilization of DIN loads^60^ and decreasing DON loads could reflect upstream N management (e.g., changing N fertilizer application timing)^55^. Conversely, MAR DIP and DOP loading significantly increased from 1985 to 2019, contrasting with conclusions from studies based on earlier data^40^. Similar to many other NGoM nutrient studies, the NGoM surface nutrient data set is dominated by samples collected in the summer months (Fig. S2).

Increases in P in the MAR watershed could come from myriad sources over time, since the watershed covers almost half the contiguous US states. Though P additions have been somewhat curtailed from wastewater treatment and industry, agriculture (i.e., pesticides, herbicides, and fertilizers) is still a sizeable source of extremely high P inputs to the MAR^81,82^. Long-lived organic P species that are used in many pesticides, herbicides, and fertilizers have the potential to persist in water and soil and act as a source of P that accumulates and is remobilized and recycled over long timescales^83^. In addition, legacy nutrients stored in soils and reservoirs continually leach into the MAR watershed, and though legacy nutrient sources have been well studied for N^13^, the magnitude and residence times for legacy P pools are harder to model and measure^14,83^.

In the MAR, although there were clear temporal trends in DON loading and all forms of P loading, there was also considerable interannual variability. While MAR DIN and DIP concentrations varied by approximately a factor of 2 interannually, MAR discharge ranged across nearly an order of magnitude over the same time period. MAR mean discharge in 2019 was by far the highest of our study period, and also contributed the highest mean annual TDN and TDP loading since 1985. Prior studies have highlighted that much of the interannual variability in MAR nutrient fluxes can be attributed to interannual variability in precipitation across the MAR watershed^55,64,75^. For example, 50 to 67% of the interannual variation in MAR N fluxes is accounted for by river discharge alone^60,84^. In turn, MAR watershed precipitation and discharge has been shown to correlate with the El Niño Southern Oscillation and the Atlantic Multidecadal Oscillation (AMO)^34,56,60^.

In addition, during high flow years in which MAR spillways (e.g., Morganza, Bonnet Carré) were opened, nutrient dynamics and location of freshwater delivery to the NGoM fundamentally change. The Bonnet Carré spillway diverts water from the Mississippi River into Lake Ponchartrain, which then connects to the NGoM, resulting in lower concentrations of suspended sediment, TDN, TDP, DIN, and DIN:DIP, and higher DON content^85^. Openings of the Bonnet Carré spillway in 1997, 2008, 2011, 2016, 2018, and 2019^86,87^, coincided with low NGoM DIN:DIP in our study. However, it is difficult to assess the relative importance of spillway openings, versus general increased flow, on nutrient trends.

Significant positive temporal correlations between MAR nutrients, MAR loading, and MAR discharge, and NGoM nutrient concentrations and ratios indicated that processes within the MAR watershed, especially influences on discharge, contributed significantly to interannual variability in NGoM surface nutrients, though with decreasing influence toward the offshore region. Similarly, Lehrter et al. (2009) found that the influence of MAR discharge on chlorophyll concentration and primary productivity diminishes across the broader shelf. In the offshore region, interannual variability in DIP concentrations remained significantly correlated to MAR discharge, MAR DIP concentrations, and MAR DIN:DIP, while interannual variability of offshore DIN concentrations was not significantly correlated to the MAR observations. Thus, links between riverine inputs and offshore DIP concentrations were stronger than for offshore DIN, consistent with greater nearshore N retention^60,88^.

As mentioned above, the rise in MAR DIN loading and consequent nearshore hypoxia in the NGoM observed from the 1950s to the 1980s^35,36,89^ seems to have stabilized during the period of this study. More recently, Karnauskas et al. (2015) suggested that an ecosystem-wide reorganization of the NGoM occurred in the mid-1990s as a result of physical ecosystem changes driven by the AMO; which was in a cool phase from the 1970s to the 1990s, and in a warm phase from the mid-1990s to at least the mid-2000s. The AMO warm phase decreased rainfall, MAR discharge, and mixed layer depth^56^. Some observations in this study may relate to the AMO because in the mid-1990s, annual mean NGoM DIN and DIP concentrations decreased from earlier values, resulting in DIN:DIP close to and below 16; especially in the offshore region, where DIN and DIP concentrations were extremely low. In the early 2000s, DIN and DIP concentrations began to rebound. If the AMO sets the stage for the processes that control nutrient distributions in the NGoM, as the AMO returns to a cool phase^90^ then we would expect DIP to again fall.

An extensive analysis of NGoM surface nutrient data from 1985 to 2012 by Cardona et al. (2016) suggested that there was an increase in surface DIN and DIP utilization from 2010 to 2011, possibly connected to the Deepwater Horizon oil spill, or to an exceptional deep winter mixed layer^31^. With the advantage of additional years of observations, the NGoM nutrient conditions from 2010 to 2012 appear to have only been a temporary shift, as the prior conditions recurred post-2013. It remains to be seen whether the long-term increase in DIP concentrations throughout the NGoM described in this study will lead to a longer-term shift in nutrients.

Nutrient concentrations influence and correlate with phytoplankton biomass, community composition, and spatial variance in chlorophyll-*a* concentrations throughout the NGoM^91,92^. In this study, we showed that surface DIP concentrations in the NGoM increased over time, while DIN did not. This increase in DIP relative to DIN has implications for nutrient limitation of phytoplankton growth and production, especially since the system has been classified as both P and N limited through time^11^. While phytoplankton in the NGoM have been described as predominantly N limited^11,31,52^, transient P limitation (and higher DIN:DIP) has been observed in spring and summer months in the MAR plume and nearshore region of the NGoM^42,70,92^. In general, N limitation typically occurs with N concentrations lower than 1 µM and N:P less than 10, while P limitation commonly is associated with N:P greater than 30^47,93,94^, though empirical results sometimes deviate from the predictions of these criteria^95^. Nevertheless, applying these nutrient ratio criteria, data in this study suggest P limitation is plausible throughout the salinity gradient in the NGoM, though the conditions for N limitation are more likely at high salinities and in the offshore region^45^. Meanwhile, the temporal increase in NGoM DIP concentrations suggests more frequent and pervasive N limitation of phytoplankton over time. P limitation at low salinities has the potential to lessen the effect of eutrophication on bottom water oxygen concentrations and reduce the incidence and strength of NGoM hypoxia^54^. Increasing excess P may therefore increase the occurrence and severity of NGoM hypoxia.

N fixation should be favored in N-limited surface waters with ample light and high DIP concentrations^96^. Given increasing likelihood of N limitation and P availability, the suitability of the NGoM for N fixation may have increased during the 1985 to 2019 time period^97^. If this trend continues, increased N fixation in the NGoM should also enhance C sequestration within the NGoM, as has been proposed for other areas affected by river plumes^98^.

Nutrients in both the MAR and the NGoM have changed over the last 35 years. In the MAR, N loading stabilized and P loading significantly increased since 1985. In the NGoM, DIP concentrations increased over time, while DIN concentrations did not. These changes were primarily driven by trends in the shelf region, as opposed to further offshore. Increases in MAR DIP loading by itself could not explain the distribution of NGoM DIP with salinity. MAR TDP loading would be sufficient to account for spatial and temporal DIP patterns in the NGoM, but only if all MAR DOP was converted to DIP by enzymatic reactions and/or photolysis. Anomalous nutrient conditions from 2010 to 2012 did not persist. Nevertheless, the increasing likelihood of N limitation and excess P availability in the NGoM has implications for phytoplankton communities, diazotrophy, and the prevalence and extent of nearshore hypoxia.

### Supplementary Information

Supplementary Information.

## Data Availability

The datasets generated during and analyzed during the current study are available from the corresponding author on reasonable request. All compiled data sources are included in the Supplementary Table S1. MAR nutrient concentration data were compiled from USGS, and nutrient loads were sourced from USGS Load Estimation (LOADEST) and Weighted Regressions on Time, Discharge, and Season method (WRTDS) USGS data (Lee, 2022) from St. Francisville, LA, USA (USGS 07373420 Hydrologic Unit 08070100).
