# Supplementary material for: Spatial and temporal variation in surface nitrate and phosphate in the Northern Gulf of Mexico over 35 years
Source: Sci Rep. 2024 Mar 27;14:7305. doi: 10.1038/s41598-024-58044-4 (PMC10973365; doi:10.1038/s41598-024-58044-4)
Supplement: Supplementary file 1 — Supplementary Information. [file 41598_2024_58044_MOESM1_ESM.pdf]

Electronic Supplementary Material

Spatial and Temporal Variation in Surface Nitrate and Phosphate in the Northern Gulf of Mexico over 35 years

Authors:

Acosta, Kailani G.[1 *], 61 Route 9W, Palisades NY 10964, kailani.acosta@columbia.edu, ORCID: 0000-0002-5216-8206

Juhl, Andrew R.[1], ORCID: 0000-0002-1575-3756, andyjuhl@ldeo.columbia.edu

Subramaniam, Ajit[1], ORCID: 0000-0003-1316-5827, ajit@ldeo.columbia.edu

Duhamel, Solange[2], ORCID: 0000-0002-8435-4695, duhamel@arizona.edu

Affiliation:

[1] Lamont-Doherty Earth Observatory, Columbia University, Palisades, NY, USA 10964

[2] Department of Molecular and Cellular Biology, University of Arizona, Tucson, AZ, USA 85721

Corresponding author information:

Kailani Acosta, kailani.acosta@columbia.edu

Supplementary Tables

Table S1 NGoM surface nutrient data sources. Data were compiled from the Biological & Chemical Oceanography Data Management Office (BCO-DMO), National Oceanic and Atmospheric Administration National Centers for Environmental Information World Ocean Database (NOAA NCEI WOD), United States Geological Survey (USGS), and Gulf of Mexico Research Initiative Information and Data Cooperative (GRIIDC). Our data set included only surface (0 to 5 m collection depth) nutrient data in the NGoM (defined as coordinates -98˚, -79˚ to 22.5˚, 31˚; see Figure 1) from 1985 to 2019, with most data collected in summer months (see Figure S1)

**Years**	**Source**	**Cruise**	**Location**	**Month**	**# of samples**	**Units**	**Properties**
1955 - 2019	USGS Lee, 2004	USGS MAR monitoring station	St. Francisville, LA	Monthly, Year round	408	Acre/ft (flow), Ton (load)	Flow, TDN load, TDP load, Temp, NO3+NO2 load, PO4, NH4, Chl a, Pigments, SiO3, pH, DO, suspended sediment, metals, pesticides
1985 - 2012	Cardona et al., 2016	GM0212, GM0303, GM306, GM0311, GM0404, GM0503, GM0509, GM0604, GM0606, GM0609, GM0704, GM0708	Shelf, Offshore	Year round	619	µM	Latitude, Longitude, Temp, Sal, Depth, NO2+NO3, NH4, PO4, Si
1985	Rabalais & Smith, 2017	LAHS 1985	LAHS	July, September	299	µM	Latitude, Longitude, Temp, Sal, Bottom Depth, Depth, NO3+NO2, PO4, NH4, Chl a, Phaeo, SiO3, pH ,DO
1986	Rabalais & Smith, 2017	LAHS 1986	LAHS	July	215	µM	Latitude, Longitude, Temp, Sal, Bottom Depth, Depth, NO3+NO2, PO4, NH4, Chl a, Phaeo, SiO3, pH ,DO
1987	Rabalais & Smith, 2017	LAHS 1987	LAHS	July	203	µM	Latitude, Longitude, Temp, Sal, Bottom Depth, Depth,

							NO3+NO2, PO4, NH4, Chl a, Phaeo, SiO3, pH ,DO
1985, 1986	Science Applications International Corp., 2002	WSK3051, Altair MX-43	Western GoM	October, January	55	µM	Latitude, Longitude, Temp, Sal, Bottom Depth, Depth, NO3+NO2, PO4, Si
1989	Murphy, 2002	Gyre 89G15	Offshore	Novemb er	10	µM	Latitude, Longitude, Temp, Sal, Depth, NO3+NO2, PO4, NH4, Si
1990	Rabalais, 2002	NECOP-90	Shelf	July, August	95	µM	Latitude, Longitude, Temp, Sal, DO, Depth, NO3+NO2, PO4, NH4, SiO3
1990	Rabalais & Smith, 2017	LATEX shelf	Shelf	July	3	µM	Latitude, Longitude, Temp, Sal, Bottom Depth, Depth, NO3+NO2, PO4, NH4, Chl a, Phaeo, SiO3
1991	Webb, 2002	Gyre WYU8126	Offshore	June	11	µM	Latitude, Longitude, Temp, Sal, Depth, NO3, PO4, Silicate
1991	Rabalais, 2002	NECOP-91	Shelf	Year round	28	µM	Latitude, Longitude, Temp, Sal, Depth, NO3+NO2, PO4, NH4, Chl a, Phaeo, SiO3, suspended sediments
1992	Rabalais, 2002	NECOP 92	Shelf	Year round	142	µM	Latitude, Longitude, Temp, Sal, Depth, NO3+NO2, PO4, NH4, Chl a, Phaeo, SiO3, suspended sediments
1992	Rabalais, 2002	NECOP 92	Shelf	April	132	µM	Latitude, Longitude, Temp, Sal, DO, Depth, NO3+NO2, PO4, NH4, Chl a, pH, SiO4
1993, 1994	Pittman, 2002	LATEX - A	Shelf	April - May	676	µmol/ L	Latitude, Longitude, Sal, Depth, DO, NO3+NO2, PO4, NH4, SiO3, chl a, urea, TSS
1993	Rabalais, 2002	NECOP 4-1993/ 7-1994	Shelf	April, July	825	µM	Latitude, Longitude, Temp, Sal, Depth, DO, NO3+NO2, PO4, NH4, chl a, phaeo, SiO3, suspended sediments
1994	Rabalais et al., 2017	LAHS 1994	LAHS	July	73	µM	Latitude, Longitude, Temp, Sal, Depth, NO3+NO2, PO4, NH4, SiO3
1994 - 2010	Parsons et al., 2014	-	Shelf	Year round	34	µM	Latitude, Longitude, Temp, Sal, Depth, NO2+NO3, NH4, PO4, SiO3, Chla, cell counts
1995	Rabalais & Smith, 2017	LAHS 1995	LAHS	July	74	µM	Latitude, Longitude, Temp, Sal, Depth, NO3+NO2, PO4, NH4, SiO3

1996	Rabalais & Smith, 2017	LAHS 1996	LAHS	July	76	µM	Latitude, Longitude, Temp, Sal, DO, Depth, NO3+NO2, PO4, NH4, SiO3
1997 - 2000	Howard, 2002	NEGOM 1 – 9 Gyre	Shelf	November, May - August	1191	µM, mg/m^3	Latitude, Longitude, Temp, Sal, Bottom Depth, Depth, DO, NO3+NO2, PO4, NH4, SiO3, Chl a, pigments
1997	Rabalais & Smith, 2017	LAHS 1997	LAHS	July	81	µM	Latitude, Longitude, Temp, Sal, DO, Depth, NO3+NO2, PO4, NH4, SiO3
1998	Rabalais & Smith, 2017	LAHS 1998	LAHS	year round	190	µM	Latitude, Longitude, Temp, Sal, Depth, NO3+NO2, PO4, NH4, SiO3
1999	Rabalais & Smith, 2017	LAHS 1999	LAHS	July	188	µM	Latitude, Longitude, Temp, Sal, Depth, NO3+NO2, PO4, NH4, SiO3
2000	Rabalais & Smith, 2017	LAHS 2000	LAHS	Year round	75	µM	Latitude, Longitude, Temp, Sal, Depth, NO3+NO2, PO4, NH4, SiO3
2000-2002	Rowe et al., 2002	NOCD 2192	Continental slope	May - June	107	µM, mg/m^3	Latitude, Longitude, Temp, Sal, Bottom Depth, Depth, DO, NO3+NO2, PO4, NH4, SiO3, urea, POC, PON, pigments
2001	Rabalais & Smith, 2017	LAHS 2001	LAHS	Year round	101	µM	Latitude, Longitude, Temp, Sal, Depth, NO3+NO2, PO4, NH4, SiO3
2002	Rabalais & Smith, 2017	LAHS 2002	LAHS	Year round	490	µM	Latitude, Longitude, Temp, Sal, Depth, NO3+NO2, PO4, NH4, SiO3
2003	Rabalais & Smith, 2017	LAHS 2003	LAHS	Year round	223	µM	Latitude, Longitude, Temp, Sal, Depth, DO, NO3+NO2, PO4, NH4, SiO3
2004	Rabalais & Smith, 2017	LAHS 2004	LAHS	Year round	240	µM	Latitude, Longitude, Temp, Sal, Depth, DO, NO3+NO2, PO4, NH4, SiO3
2004	Cardona et al., 2016	LC0401, LC0402, LC0403	Shelf	January - March	95	µM	Latitude, Longitude, Temp, Sal, Depth, NO3+NO2, PO4, NH4, chl a
2004	Cardona et al., 2016	SEAMAP	Shelf	May, June	99	µM	Latitude, Longitude, Temp, Sal, Bottom Depth, Depth, NO3+NO2, PO4, NH4, chl a
2004	Cardona et al., 2016	GM0503 GM0404	Shelf	May, June	98	µM	Latitude, Longitude, Temp, Sal, Bottom Depth, Depth, NO3+NO2, PO4, NH4, chl a

2005	Rabalais & Smith, 2017	LAHS 2005	LAHS	Year round	259	µM	Latitude, Longitude, Temp, Sal, Depth, NO3+NO2, SiO3, PO4, NH4
2006	Rabalais & Smith, 2017	LAHS 2006	LAHS	Year round	164	µM	Latitude, Longitude, Temp, Sal, Depth, NO3+NO2, SiO3, PO4, NH4
2007	Rabalais & Smith, 2017	LAHS 2007	LAHS	Year round	232	µM	Latitude, Longitude, Temp, Sal, Depth, NO3+NO2, SiO3, PO4, NH4, Chl a
2007	Langdon, 2010	GOMECC 2007	Shelf, offshore	July, August	95	µmol/kg	Latitude, Longitude, Temp, Sal, Bottom Depth, Depth, DO, NO3+NO2, SiO3, PO4, NH4, Chl a, Silicate, CFCs, TOC, POC, DOC
2008	Cardona et al., 2016	MAGMIX	LAHS	May, November	99	µM	Latitude, Longitude, Temp, Sal, Bottom Depth, Depth, NO3+NO2, PO4, NH4, chl a
2008	Rabalais & Smith, 2017	LAHS 2008	LAHS	Year round	206	µM	Latitude, Longitude, Temp, Sal, Bottom Depth, Depth, NO3+NO2, PO4, NH4, SiO3
2009	Rabalais & Smith, 2017	LAHS 2009	LAHS	Year round	208	µM	Latitude, Longitude, Temp, Sal, Bottom Depth, Depth, NO3+NO2, PO4, NH4, SiO3
2010	Rabalais & Smith, 2017	LAHS 2010	LAHS	Year round	213	µM	Latitude, Longitude, Temp, Sal, Bottom Depth, Depth, NO3+NO2, PO4, NH4, SiO4, Chl a
2010	Montoya, 2016	OC468, EN496	Shelf, offshore	August - September	85	µM	Latitude, Longitude, Temp, Sal, Density, Depth, NO3+NO2, PO4, DO, N*
2010 - 2011	Shiller, 2014	R/V Pelican 2010	Deepwater Horizon area	May, October	29	µM	Latitude, Longitude, Temp, Sal, Depth, Bottom Depth, NO3+NO2, TDN, PO4, NH4, SiO4, O2, Chl a, Cs, Mo, Ra, Ba, V, Cr, Fe, Ni, Cu, Mn, Co, PAHs
2010 - 2013	Joye, 2016	EN510, EN515, EN528, FK006b, WS1010	GC600, Taylor Energy area	May - December	26	µM	Latitude, Longitude, Temp, Sal, Depth, NH4, NO3+NO2, TDN, TDP, DOC, DON, DOP, PO4, DO, pH, cell counts,
2010, 2011	Cardona et al., 2016	GOMEX2011, EN496, CH0711	Shelf, offshore	July	976	µM	Latitude, Longitude, Temp, Sal, Depth, DO, CDOM, NO2+NO3,

							NH4, PO4, SiO2, Chl a, cell counts
2011	Rabalais & Smith, 2017	LAHS 2011	LAHS	April - Septemb er	171	µM	Latitude, Longitude, Temp, Sal, Bottom Depth, Depth, NO3+NO2, PO4, NH4, SiO3
2011 - 2012	Daly, 2014	BE0412, WB0111, WB0211, WB0212, WB0511, WB0512, WB0611, WB0911, WB1111, WB1210	West Florida Shelf	January - Novemb er	761	µM	Temp, Depth, Urea, NO2+NO3, NH4, PO4, TDN, TDP, Si
2011, 2015, 2017, 2018	Howe et al., 2020	PE18-23, PE17-24, WBII 03 2015, WS1114	Offshore	April, June, Novemb er	8	µM	Latitude, Longitude, Sal, Depth, NO3+NO2, d18O
2012	Rabalais, 2017	LAHS 2012	LAHS	May - Septemb er	136	µM	Latitude, Longitude, Temp, Sal, Bottom Depth, Depth, NO3+NO2, PO4, NH4, SiO4
2012	Kostka et al., 2014	WB-1306CT7	Seeps (Peanut Seep, A, B, C)	Septemb er - October	4	µM	Latitude, Longitude, Temp, Sal, Depth, PP, NO2+NO3, NH4, PO4, SiOH4, DO, Turbidity, Density, Chl a, CH4
2012	Montoya, 2019	EN509	Shelf, Offshore	May-June	38	µM	Latitude, Longitude, Temp, Sal, Density, Depth, NO3+NO2, PO4, DO, N*
2012	Stewart et al., 2015	CH0212	Shelf	July - August	64	µM	Latitude, Longitude, Depth, Temp, Sal, NO3+NO2
2012 - 2013	Sutor, 2015	Walton Smith	Offshore	April	895	µM	Latitude, Longitude, Depth, Temp, Sal, Density, NH4, N+N, NO2
2012 - 2014	Daly, 2015	R/V Bellows	West Florida Shelf	April - Novemb er	930	µM	Temp, Depth, Urea, NO2+NO3, NH4, PO4, TDN, TDP, Si
2013	Montoya, 2016	R/V Arcadiana, R/V Weatherbird	NGoM Hercules Spill Response	July - August	9	µM	Latitude, Longitude, Sal, Density, Depth, NH4, NO3+NO2, TDN, TDP, DOC, DON, DOP, PO4, DO, pH, cell counts
2013	Rabalais & Smith, 2017	LAHS 2013	LAHS	July	89	µM	Latitude, Longitude, Temp, Sal, Bottom Depth, Depth, NO3+NO2, PO4, NH4, SiO4
2014	Roberts, 2016	R/V Pelican PE-15-01 LUMCON	Plume to offshore NGoM	July	82	µM	Latitude, Longitude, Temp, Depth, PP, NO2+NO3, NH4, PO4, SiO2, DO, Chl a, Phaeo

2014	Rabalais, 2017	LAHS 2014	LAHS	July	178	µM	Latitude, Longitude, Temp, Sal, Bottom Depth, Depth, NO3+NO2, PO4, NH4, SiO3
2015	Barbero et al., 2016	WS1509	OAP 2015	Septemb er	30	µmol/ kg	Latitude, Longitude, Temp, Sal, Bottom Depth, Depth, NO3+NO2, PO4, DIC, Silicate, pH
2015, 2016	Krause & Acton, 2018	PTS01	Shelf	October, Novemb er	48	ug/L	Latitude, Longitude, Depth, Bottom Depth, POC, PON, Chl a
2015	Shiller, 2017	R/V Pt. Sur CONCORDE BCS 2015, 2016	Mississippi Bight	October, Novemb er,Febru ary, July	102	umol/ kg	Latitude, Longitude, Temp, Sal, Depth, NO3+NO2, PO4, NH4, SiO4, O2, Chl a
2015, 2016	Joye, 2018; Zhuang et al., 2018	EN559, EN586		May - June	10	µmol/ L	Latitude, Longitude, Sal, Depth, NO3, NH4, PO4, DOC, Chl a
2016	Caffrey, 2017		Pensacola shelf	July	73	TN (ug N/L) Si (mg/L) TKN/ TP (mg N/ mg P)	Total Nitrate/Nitrite, Total Kjeldahl Nitrogen (FIA), Total Phosphorus (FIA), Silica
2016	Rabalais, 2019	LAHS 2016	LAHS	August	21	µM	Latitude, Longitude, Temp, Sal, Bottom Depth, Depth, NO3+NO2, PO4, NH4, SiO3
2016	Joye, 2019	EN586	NGoM	July - August	310	µM	Latitude, Longitude, Sal, Depth, NH4, NO3+NO2, TDN, TDP, DOC, DON, DOP, PO4, DO
2016, 2017	Krause et al., 2019	CLASiC2016, CLASiC2017	Shelf	May, August, Septemb er	59	µM	Latitude, Longitude, Temp, Sal, Oxygen, Depth, Bottom Depth, NO3, NO2, NH4, PO4, Silicate, pheophytin, Chl a,
2017	Rabalais, 2020	LAHS 2017	LAHS	July	93	µM	Latitude, Longitude, Temp, Sal, Bottom Depth, Depth, NO3+NO2, PO4, NH4, SiO4
2017	Olascoaga, M.J., 2019	GOMECC3	Shelf, Offshore	July, August	1631	µmol/ kg	Latitude, Longitude, Temp, Sal, Bottom Depth, Depth, DO, NO3+NO2, SiO3, PO4, NH4, Chl a, Silicate, CFCs, TOC, POC, DOC

2017	Kamalanathan et al., 2019	PS1809	Shelf	September, October	126	µmol/L, ug/L	Latitude, Longitude, Temp, Sal, Depth, Bottom Depth, NO3, NO2, NH4, PO4, Urea
2018	Rabalais, 2020	LAHS 2018	LAHS	July	51	µM	Latitude, Longitude, Temp, Sal, Bottom Depth, Depth, DO, NO3+NO2, PO4, NH4, SiO4
2018, 2019	This Study	EN620, EN642	Offshore, shelf	July, August	89	µM	Latitude, Longitude, Sal, Density, Depth, NO3+NO2, PC, PN, PP, TDN, TDP, DON, DOP, PO4, DO, cell counts, pigments
2019	Barbero et al., 2019	WS19119, WS19210,WS19266	OAP 19	May - October	20	µmol/kg	Latitude, Longitude, Temp, Sal, Bottom Depth, Depth, NO3+NO2, PO4, DIC, Silicate, pH

References

Barbero, L.,Wanninkhof, R., & Pierrot, D. (2016). Dissolved inorganic carbon, total alkalinity, pH, nutrients, and other variables collected from surface discrete observations using Niskin bottle and other instruments from R/V F. G. Walton Smith in the west coast of Florida within Gulf of Mexico from 2015-09-21 to 2015-09-25 (NCEI Accession 0157025). NOAA National Centers for Environmental Information. Dataset. https://doi.org/10.7289/v5ws8r98.

Barbero, L.,Wanninkhof, R., & Pierrot, D. (2019). Dissolved inorganic carbon, total alkalinity, pH, nutrients, and other variables collected from surface discrete observations using Niskin bottle and other instruments during the R/V F. G. Walton Smith cruise WS19028 (EXPOCODE 33WA20190128) in the west coast of Florida, Gulf of Mexico from 2019-01-28 to 2019-02-01 (NCEI Accession 0188977). [indicate subset used]. NOAA National Centers for Environmental Information. Dataset. https://doi.org/10.25921/dnxy-k985.

Caffrey, J. (2017). Surface and Bottom Water Nutrient Data July 2016, Pensacola Shelf. Distributed by: Gulf of Mexico Research Initiative Information and Data Cooperative (GRIIDC), Harte Research Institute, Texas A&M University–Corpus Christi. doi:10.7266/N7DZ06QP

Cardona, Y., Bracco, A., Villareal, T. A., Subramaniam, A., Weber, S. C., & Montoya, J. P. (2016). Highly variable nutrient concentrations in the Northern Gulf of Mexico. *Deep-Sea Research Part II: Topical Studies in Oceanography*, *129*, 20–30. https://doi.org/10.1016/j.dsr2.2016.04.010

Daly, K. (2014). Nutrient concentrations for the northeastern Gulf of Mexico and west Florida shelf, July 2010 – May 2012.. Distributed by: Gulf of Mexico Research Initiative Information and Data Cooperative (GRIIDC), Harte Research Institute, Texas A&M University–Corpus Christi. doi:10.7266/N7R78C41

Daly, K. (2015). Nutrient concentrations for the northeastern Gulf of Mexico and west Florida shelf: June 2012 – August 2014. Distributed by: Gulf of Mexico Research Initiative Information and Data Cooperative (GRIIDC), Harte Research Institute, Texas A&M University–Corpus Christi. doi:10.7266/N7G73BMH

Howard, M. K. (2002). Chemical, current meter, and other data from current meter, bottle, XBT, and CTD casts in the Gulf of Mexico as part of the Northeastern Gulf of Mexico Physical Oceanographic Program: Chemical Oceanography and Hydrography Study (NEGOM) project, 1997-11-16 to 2000-08-08 (NCEI Accession 0000703). NOAA National Centers for Environmental Information. https://www.ncei.noaa.gov/archive/accession/0000703.

Joye, S. (2016). Water column 16S rRNA libraries, geochemical data, and process rate data collected from the Northern Gulf of Mexico before, during, and after the Deepwater Horizon Event March 2010 - November 2012. Distributed by: Gulf of Mexico Research Initiative Information and Data Cooperative (GRIIDC), Harte Research Institute, Texas A&M University–Corpus Christi. doi:10.7266/N7DV1GWK

Joye, S. (2018). Methanol and methylamine oxidation rate measurements, geochemistry, and bacterial production in the northern Gulf of Mexico water column, May 2015 to August 2016. Distributed by: Gulf of Mexico Research Initiative Information and Data Cooperative (GRIIDC), Harte Research Institute, Texas A&M University–Corpus Christi. doi:10.7266/N7SF2TPP

Joye, S. (2019). Geochemical data collected from R/V Endeavor cruise EN585 at the Orca Basin in the Gulf of Mexico on 2016-07-29. Distributed by: Gulf of Mexico Research Initiative Information and Data Cooperative (GRIIDC), Harte Research Institute, Texas A&M University–Corpus Christi. doi:10.7266/n7-3bp5-sb94

Kamalanathan, M., Mapes, S., Hillhouse, J., Claflin, N., Campbell, D., & Quigg, A. (2019). How does oil exposure affect centric diatom Thalassiosira pseudonana?. Distributed by: Gulf of Mexico Research Initiative Information and Data Cooperative (GRIIDC), Harte Research Institute, Texas A&M University–Corpus Christi. doi:10.7266/BSYXMGD0

Kostka J. E., Teske AP, Joye S. B., Head I.M. (2014). The metabolic pathways and environmental controls of hydrocarbon biodegradation in marine ecosystems. *Frontiers in Microbiology* (*5*)471. doi: 10.3389/fmicb.2014.00471.

Krause, J.W. & Acton, S. (2018). Size-fractionated nitrate uptake and primary productivity on the Mississippi-Alabama Shelf, March to July, 2016. Distributed by: Gulf of Mexico Research Initiative Information and Data Cooperative (GRIIDC), Harte Research Institute, Texas A&M University–Corpus Christi. doi:10.7266/N74B2ZVN

Krause, J.W., Thamatrakoln, K., Cole, L., & Acton, S. (2019). Seasonal effects on microplankton response to oil perturbation on Mississippi-Alabama shelf, Gulf of Mexico from 2016-01-24 to 2017-03-16. Distributed by: Gulf of Mexico Research Initiative Information and Data Cooperative (GRIIDC), Harte Research Institute, Texas A&M University–Corpus Christi. doi:10.7266/n7-w8zg-tj49

Langdon, C. (2010). Temperature, salinity, nutrients, freons, oxygen, currents (ADCP), underway and other measurements collected in the Gulf of Mexico and Atlantic as part of the Gulf of Mexico and East Coast Carbon Cruise (GOMECC) 2007 (NCEI Accession 0066603). NOAA National Centers for Environmental Information. Dataset. https://www.ncei.noaa.gov/archive/accession/0066603.

Lee, C. (2022). Nutrient loads to the Gulf of Mexico produced by the USGS National Water Quality Network, 1968-2021: U.S. Geological Survey, https://doi.org/10.5066/P9G0EEUE

Montoya, J. (2016). Hercules Spill Response Cruises, Water Chemistry. Louisiana South Timbalier Block 220, 27-30 July (R/V Acadiana) and 25 August (R/V Weatherbird) 2013. Distributed by: Gulf of Mexico Research Initiative Information and Data Cooperative (GRIIDC), Harte Research Institute, Texas A&M University–Corpus Christi. doi:10.7266/N7125QNH

Montoya, J. (2016). Particle and zooplankton sample elemental (C, N) and isotopic (13C, 15N) data from Cruise OC468, EN496, Louisiana, Mississippi, Alabama Self Break, August and September, 2010. Distributed by: Gulf of Mexico Research Initiative Information and Data Cooperative (GRIIDC), Harte Research Institute, Texas A&M University–Corpus Christi. doi:10.7266/N7X06521

Montoya, J. (2019). Particle and zooplankton sample elemental (C,N) and isotopic (13C, 15N) data, Cruise EN509, northern Gulf of Mexico, May 26 - June 19, 2012. Distributed by: Gulf of Mexico Research Initiative

Information and Data Cooperative (GRIIDC), Harte Research Institute, Texas A&M University–Corpus Christi. doi:10.7266/N7NK3C2R

Murphy, D. (2002). Cloud amount/frequency, NITRATE and other data from GYRE in the Gulf of Mexico from 1989-11-11 to 1989-11-18 (NCEI Accession 9000025). NOAA National Centers for Environmental Information. https://www.ncei.noaa.gov/archive/accession/9000025.

Olascoaga, M.J. (2019). Drifters released during the Gulf of Mexico Ecosystems and Carbon Cycle (GOMECC-3) cruise in the Gulf of Mexico from 2017-07-22 to 2017-09-17. Distributed by: Gulf of Mexico Research Initiative Information and Data Cooperative (GRIIDC), Harte Research Institute, Texas A&M University–Corpus Christi. doi:10.7266/N7G44NW5

Ozhan, K., Parsons, M. L., & Bargu, S. (2014). How Were Phytoplankton Affected by the Deepwater Horizon Oil Spill? *BioScience*, *64*(9), 829–836. https://doi.org/10.1093/BIOSCI/BIU117

Pittman, R.V. (2002). BAROMETRIC PRESSURE and Other Data from GYRE From Gulf of Mexico from 1993-10-28 to 1993-11-03 (NCEI Accession 9400017). NOAA National Centers for Environmental Information. Dataset. https://www.ncei.noaa.gov/archive/accession/9400017.

Rabalais, N. N. (2002). Chemical, zooplankton, and phytoplankton data from CTD and other instruments in the Mississippi River and Gulf of Mexico as part of the Nutrient Enhanced Coastal Ocean Productivity (NECOP) project, from 1985-07-15 to 1993-05-12 (NCEI Accession 9800129). NOAA National Centers for Environmental Information. Dataset. https://www.ncei.noaa.gov/archive/accession/9800129.

Rabalais, N. N. (2017). Physical (Hydrography), chemical (CTD), and biological (Water Quality) processes of the Texas-Louisiana continental shelf, 2012 (NCEI Accession 0162101). NOAA National Centers for Environmental Information. Dataset. https://www.ncei.noaa.gov/archive/accession/0162101.

Rabalais, N. N. (2017). Physical (Hydrography), chemical (CTD), and biological (Water Quality) processes of the Texas-Louisiana continental shelf, 2014 (NCEI Accession 0161219). NOAA National Centers for Environmental Information. Dataset. https://www.ncei.noaa.gov/archive/accession/0161219.

Rabalais, N. N. (2019). Water temperature, salinity, and other physical, chemical, and biological parameters taken by CTD and multi parameter water quality sonde on board of research vessel Pelican on the Texas-Louisiana continental shelf, Gulf of Mexico from 2016-06-14 2016-10-26 (NCEI Accession 0205845). NOAA National Centers for Environmental Information. Dataset. https://doi.org/10.25921/5903-cp08.

Rabalais, N. N. (2020). Physical (hydrography), chemical (CTD), and biological (water quality) processes of the Texas-Louisiana continental shelf, 2017 (NCEI Accession 0208325). NOAA National Centers for Environmental Information. Dataset. https://www.ncei.noaa.gov/archive/accession/0208325.

Rabalais, N. N. (2020). Physical (hydrography), chemical (CTD), and biological (water quality) processes of the Texas-Louisiana continental shelf, 2018 (NCEI Accession 0219157). NOAA National Centers for Environmental Information. Dataset. https://www.ncei.noaa.gov/archive/accession/0219157.

Rabalais, N. N., & Smith, L. (2017). Bottom-water area of Louisiana-Texas continental shelf hypoxia 1985-2014. Distributed by: Gulf of Mexico Research Initiative Information and Data Cooperative (GRIIDC), Harte Research Institute, Texas A&M University–Corpus Christi. doi:10.7266/N7GF0RKZ

Rabalais, N. N., Turner, R. E., & Wiseman, W. J.., Jr. (2017). Hydrographic and chemical water parameters collected by CTD and other instruments from the Pelican and the Tommy Munro in coastal waters of Louisiana from 1994-07-24 to 1997-07-29 (NCEI Accession 0164298). NOAA National Centers for Environmental Information. Dataset. https://www.ncei.noaa.gov/archive/accession/0164298.

Roberts, B. (2016). Dissolved inorganic nutrients, pigments, and dissolved oxygen concentrations collected during R/V Pelican cruise July 7-10 2014, northern Gulf of Mexico near DeSoto Canyon, PE15-01. Distributed by: Gulf of Mexico Research Initiative Information and Data Cooperative (GRIIDC), Harte Research Institute, Texas A&M University–Corpus Christi. doi:10.7266/N7BZ63Z7

Rowe, G. T., Cruz Kaegi, M.E., Morse, J. W., Boland, G. S., & Escobar Briones, E.G. (2002). Sediment Community Metabolism Associated with Continental Shelf Hypoxia, Northern Gulf of Mexico. *Estuaries*, *25*(6), 1097–1106. http://www.jstor.org/stable/1353154

Science Applications International Corporation. (2002). Current meter data from moored current meter casts in the Gulf of Mexico as part of the Gulf of Mexico Physical Oceanography (GMPO) project, from 1983-01-26 to 1985-01-01 (NCEI Accession 8600064). NOAA National Centers for Environmental Information. Dataset. https://www.ncei.noaa.gov/archive/accession/8600064.

Shiller, A.M. (2014). Dissolved trace metal and ancillary data (including nutrients and dissolved oxygen) in the vicinity of the Deepwater Horizon blowout, May 2010-October 2011.. Distributed by: Gulf of Mexico Research Initiative Information and Data Cooperative (GRIIDC), Harte Research Institute, Texas A&M University–Corpus Christi. doi:10.7266/N7MS3QQ5

Shiller, A.M. (2017). Geochemical CTD/rosette data for cruise PTS01 in the Mississippi Bight, Fall 2015. Distributed by: Gulf of Mexico Research Initiative Information and Data Cooperative (GRIIDC), Harte Research Institute, Texas A&M University–Corpus Christi. doi:10.7266/N7F769NC

Stewart, P. S., Feng, J., Kimptom, L. S., Griffiths, I.M., & Stone, H.A. (2015). Dataset supporting the publication "Stability of a bi-layer free film: simultaneous or individual rupture events?". Distributed by: Gulf of Mexico Research Initiative Information and Data Cooperative (GRIIDC), Harte Research Institute, Texas A&M University–Corpus Christi. doi:10.7266/N79Z92V9

Sutor, M. (2015). Inorganic nutrients in Gulf of Mexico, spring 2012 and spring 2013. Distributed by: Gulf of Mexico Research Initiative Information and Data Cooperative (GRIIDC), Harte Research Institute, Texas A&M University–Corpus Christi. doi:10.7266/N77S7KQM

van Hooidonk, R. (2022). Modeled ocean acidification data in the Gulf of Mexico and wider Caribbean using satellites and climate model data for the Ocean Acidification Products for the Gulf of Mexico and East Coast project from 2014-01-01 to 2020-12-31 (NCEI Accession 0245950). NOAA National Centers for Environmental Information. Dataset. https://doi.org/10.25921/tt1c-dx53.

Webb, E. (2002). Cloud amount/frequency, NITRATE and other data from GYRE in the Gulf of Mexico from 1991-03-02 to 1991-03-09 (NCEI Accession 9100092). NOAA National Centers for Environmental Information. Dataset. https://www.ncei.noaa.gov/archive/accession/9100092.

Zhuang, G. C., Montgomery, A., Sibert, R. J., Rogener, M. K., Samarkin, V. A., & Joye, S. B. (2018). Effects of pressure, methane concentration, sulfate reduction activity, and temperature on methane production in surface sediments of the Gulf of Mexico. *Limnology and Oceanography*, *63*(5), 2080–2092. https://doi.org/10.1002/LNO.10925

Table S2 Summary statistics for annual mean N and P loading from the MAR into the NGoM. The corresponding plots are shown in Figure 3. Linear regression equations, r^2, p values, and n for mean annual N and P loading into the NGoM from 1985 to 2019. Significant regressions are shaded green. Based on USGS data (Lee, 2022)

**N**	**Regression Equation**	r^2	***p* value**	***n***	**P**	**Regression Equation**	r^2	***p* value**	***n***
TN	$y = 1.6 \times 10^9 - 2.8 \times 10^6x$	-0.08	0.65	34	TP	$y = 1.4 \times 10^8 + 1.6 \times 10^6x$	0.44	0.009	34
DIN	$y = 9.7 \times 10^8 + 3.4 \times 10^6x$	0.05	0.74	34	DIP	$y = 3.6 \times 10^7 + 7.6 \times 10^5x$	0.48	0.004	34
DON	$y = 6.4 \times 10^8 - 6.2 \times 10^6x$	-0.43	0.01	34	DOP	$y = 1.0 \times 10^8 + 8.8 \times 10^5x$	0.31	0.05	34

Table S3 Regression results to test for temporal trends in mean annual nutrient concentrations (DIN, DIP, and DIN:DIP) for each regional subset of the NGoM from 1985 to 2019; hypoxic region, shelf region, offshore region (see Figure S5). Entire data set annual mean nutrient concentrations are shown in Figure 2

Region	Nutrient (μM)	Regression Equation	r^2	*p* value	*n*
**Hypoxic**	[DIN]	y = 5.2 – 0.06x	0.04	0.30	32
**Hypoxic**	[DIP]	y = 0.3 + 0.01x	0.19	0.012	31
**Hypoxic**	DIN:DIP	y = 10 – 0.15x	0.04	0.24	32
**Shelf**	[DIN]	y = 4.8 – 0.04x	0.02	0.45	32
**Shelf**	[DIP]	y = 0.33 + 0.01x	0.16	0.023	31
**Shelf**	DIN:DIP	y = 9.7 – 0.14x	0.04	0.27	32
**Offshore**	[DIN]	y = 4.5 – 0.08x	0.02	0.52	32
**Offshore**	[DIP]	y = 0.13 – 0.003x	0.03	0.43	32
**Offshore**	DIN:DIP	y = 4.4 + 0.01x	0.11	0.87	32

Table S4 Correlation tables for annual mean MAR nutrient concentrations, discharge, and loading vs. NGoM annual mean nutrient concentrations. Annual mean nutrient concentrations are calculated using data from St. Francisville, LA, and discharge values are from the MAR combined flow into the NGoM in m^3 (Figure S3). Pearson correlation coefficients (*r*) and *p* values shown for the entire NGoM dataset, and each of three regional subsets of the data. Significant *p*-values < 0.05 are shaded green. Because of excluded NGoM data in 1988 and 2014, *n* = 32 for all correlations. Based on USGS data (Lee, 2022)

Region	Annual Mean Nutrient	MAR discharge	MAR DIN load	MAR [DIN]	MAR DIP load	MAR [DIP]	MAR DIN:DIP
**Entire NGoM**	NGoM DIN	*r* = 0.32 *p* = 0.04	*r* = 0.48 *p* = 0.002	*r* = 0.49 *p* = 0.001	*r* = 0.37 *p* = 0.02	*r* = 0.49 *p* = 0.001	*r* = 0.42 *p* = 0.01
**Entire NGoM**	NGoM DIP	*r* = 0.45 *p* = 0.003	*r* = 0.53 *p* = 0.004	*r* = 0.52 *p* = 0.001	*r* = 0.57 *p* < 0.001	*r* = 0.65 *p* < 0.001	*r* = 0.38 *p* = 0.02
**Entire NGoM**	NGoM DIN:DIP	*r* = 0.26 *p* = 0.10	*r* = 0.44 *p* = 0.004	*r* = 0.44 *p* = 0.005	*r* = 0.37 *p* = 0.02	*r* = 0.44 *p* = 0.004	*r* = 0.33 *p* = 0.04
**Hypoxic Region**	Hypoxic DIN	*r* = 0.32 *p* = 0.05	*r* = 0.50 *p* = 0.001	*r* = 0.49 *p* = 0.001	*r* = 0.36 *p* = 0.02	*r* = 0.49 *p* = 0.001	*r* = 0.45 *p* = 0.004
**Hypoxic Region**	Hypoxic DIP	*r* = 0.41 *p* = 0.01	*r* = 0.52 *p* = 0.001	*r* = 0.50 *p* = 0.001	*r* = 0.53 *p* < 0.001	*r* = 0.62 *p* < 0.001	*r* = 0.39 *p* = 0.02
**Hypoxic Region**	Hypoxic DIN:DIP	*r* = 0.27 *p* = 0.10	*r* = 0.44 *p* = 0.004	*r* = 0.43 *p* = 0.005	*r* = 0.35 *p* = 0.03	*r* = 0.43 *p* = 0.005	*r* = 0.34 *p* = 0.03
**Shelf**	Shelf DIN	*r* = 0.33 *p* = 0.04	*r* = 0.48 *p* = 0.002	*r* = 0.48 *p* = 0.002	*r* = 0.36 *p* = 0.02	*r* = 0.50 *p* = 0.001	*r* = 0.43 *p* = 0.01
**Shelf**	Shelf DIP	*r* = 0.43 *p* = 0.01	*r* = 0.52 *p* = 0.001	*r* = 0.50 *p* = 0.001	*r* = 0.55 *p* < 0.001	*r* = 0.63 *p* < 0.001	*r* = 0.38 *p* = 0.02
**Shelf**	Shelf DIN:DIP	*r* = 0.26 *p* = 0.10	*r* = 0.44 *p* = 0.005	*r* = 0.43 *p* = 0.006	*r* = 0.36 *p* = 0.02	*r* = 0.44 *p* = 0.005	*r* = 0.33 *p* = 0.04
**Off shore**	Offshore DIN	*r* = 0.12 *p* = 0.45	*r* = 0.12 *p* = 0.44	*r* = 0.09 *p* = 0.60	*r* = 0.05 *p* = 0.77	*r* = 0.06 *p* = 0.71	*r* = 0.19 *p* = 0.24
**Off shore**	Offshore DIP	*r* = 0.43 *p* = 0.01	*r* = 0.27 *p* = 0.09	*r* = 0.30 *p* = 0.06	*r* = 0.23 *p* = 0.15	*r* = 0.33 *p* = 0.04	*r* = 0.28 *p* = 0.01
**Off shore**	Offshore DIN:DIP	*r* = 0.41 *p* = 0.01	*r* = 0.36 *p* = 0.02	*r* = 0.36 *p* = 0.02	*r* = 0.36 *p* = 0.02	*r* = 0.41 *p* = 0.01	*r* = 0.25 *p* = 0.11

Table S5 Correlations of annual mean nutrient concentrations for NGoM shelf region vs. offshore region. Table shows Pearson correlation coefficients (*r*) and *p* values. Correlation analyses were not conducted separately for the hypoxic region because it is a subset of the shelf region and therefore included in the shelf data. Significant values are shaded green

Region	Shelf [DIN]	Shelf [DIP]	Shelf (DIN:DIP)
**Offshore [DIN]**	$r = 0.07$<br>$p = 0.67$	$r = 0.13$<br>$p = 0.44$	$r = 0.05$<br>$p = 0.76$
**Offshore[DIP]**	$r = 0.28$<br>$p = 0.07$	$r = 0.24$<br>$p = 0.13$	$r = 0.04$<br>$p = 0.82$
**Offshore (DIN:DIP)**	$r = 0.32$<br>$p = 0.04$	$r = 0.36$<br>$p = 0.02$	$r = 0.04$<br>$p = 0.83$

Table S6 Results of correlation analyses comparing annual area of hypoxic bottom water to mean annual NGoM surface nutrient concentrations. Pearson correlation coefficients (*r*) and *p* values presented for (a) MAR nutrient loading, (b) the entire NGoM, and for 3 NGoM subregions (c – e). The annual area of hypoxic bottom water is from Rabalais and Turner, (2019) and annual mean nutrient data are shown in Figures 3 and S4 with accompanying summary statistics listed in Table S5 ($n = 32$). Significant values < 0.05 are shaded green

Annual hypoxic area vs.	Correlation Coefficient (Pearson's *r*)	*p* value
**a MAR nutrient loading**		
**vs. mean annual DIN load**	0.68	< 0.0001
**vs. mean annual DON load**	0.34	0.03
**vs. mean annual TN load**	0.59	< 0.0001
**vs. mean annual DIP load**	0.69	< 0.0001
**vs. mean annual DOP load**	0.41	0.01
**vs. mean annual TP load**	0.52	0.001
**b Entire NGoM dataset**		
**vs. mean annual [DIN]**	0.33	0.04
**vs. mean annual [DIP]**	0.33	0.04
**vs. mean annual DIN:DIP**	0.41	0.01
**c Hypoxic region**		
**vs. mean annual [DIN]**	0.34	0.03
**vs. mean annual [DIP]**	0.31	0.05
**vs. mean annual DIN:DIP**	0.41	0.01
**d Shelf region**		
**vs. mean annual [DIN]**	0.34	0.03
**vs. mean annual [DIP]**	0.31	0.05
**vs. mean annual DIN:DIP**	0.41	0.01
**e Offshore region**		
**vs. mean annual [DIN]**	-0.07	0.65
**vs. mean annual [DIP]**	-0.09	0.59
**vs. mean annual DIN:DIP**	0.21	0.20

Table S7 Regression results to test for salinity trends in NGoM surface nutrients by regional subset 1985–2019: (a) hypoxic ln[DIN]; (b) hypoxic ln[DIP]; (c) hypoxic ln(DIN:DIP); (d) shelf ln[DIN]; (e); shelf ln[DIP]; (f) shelf ln(DIN:DIP); (g) offshore ln[DIN]; (h) offshore ln[DIP]; (i) offshore ln(DIN:DIP) – NGoM nutrient concentrations vs. salinity for all regions combined are shown in Figure 4. The corresponding regional plots are shown in Figure S5a-i. Significant regressions are shaded green

	**Region**	**Nutrient (μM)**	**Regression Equation**	r^2	***p* value**	***n***
**a**	Hypoxic	ln[DIN]	y = -0.15x + ln(102.41)	0.25	< 0.0001	5671
**b**	Hypoxic	ln[DIP]	y = -0.07x + ln(2.19)	0.14	< 0.0001	5455
**c**	Hypoxic	ln(DIN:DIP)	y = -0.11x + ln(46.78)	0.001	0.02	5414
**d**	Shelf	ln[DIN]	y = -0.18x + ln(102.41)	0.26	< 0.0001	6313
**e**	Shelf	ln[DIP]	y = -0.07x + ln(2.19)	0.17	< 0.0001	6024
**f**	Shelf	ln(DIN:DIP)	y = -0.11x + ln(46.78)	0.001	0.01	5981
**g**	Offshore	ln[DIN]	y = -0.19x + ln(102.41)	0.34	< 0.0001	1428
**h**	Offshore	ln[DIP]	y = -0.10x + ln(2.19)	0.22	< 0.0001	1452
**i**	Offshore	ln(DIN:DIP)	y = -0.09x + ln(46.78)	0.03	< 0.0001	1392

Supplementary Figures

Figure S1

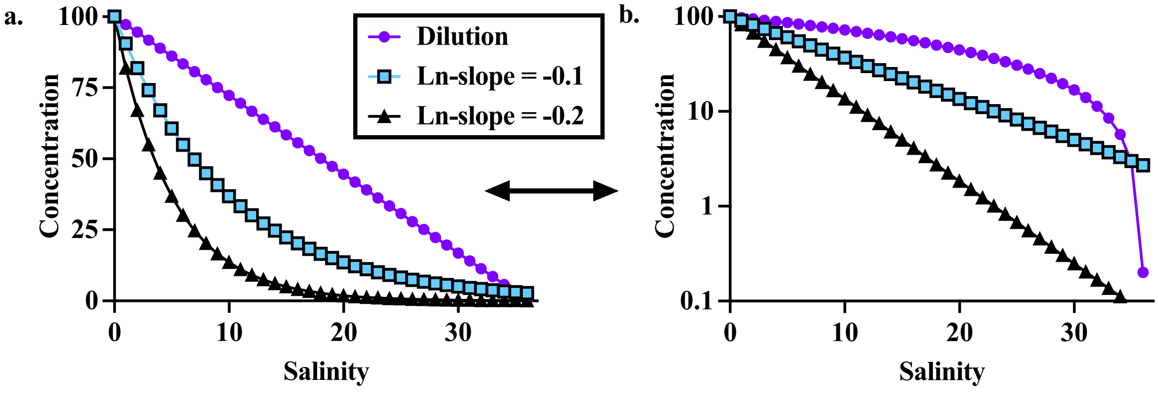

Fig. S1 Nutrient concentration vs. salinity plots for a hypothetical nutrient with a concentration at zero salinity of 100 (in arbitrary units). The same underlying data is plotted on both (a.) and (b.) though in (b.) the y-axis is log-scale (as in Fig. 4). The dilution-only mixing function (purple dots) is calculated by assuming that the nutrient declines in proportion to increasing salinity, resulting in a line in (a.) and a curve in (b.). The blue and black data points in both plots were calculated using the linear equations $\ln(y) = -0.1x + \ln(100)$, and $\ln(y) = -0.2x + \ln(100)$. These figures demonstrate that the linear mixing functions used in this study should appear curved on semi-log plots. In addition, the figures exemplify why an increasing slope of ln-transformed nutrient vs. salinity relationships primarily reflects higher relative loss of the nutrient at low salinities

Fig S2

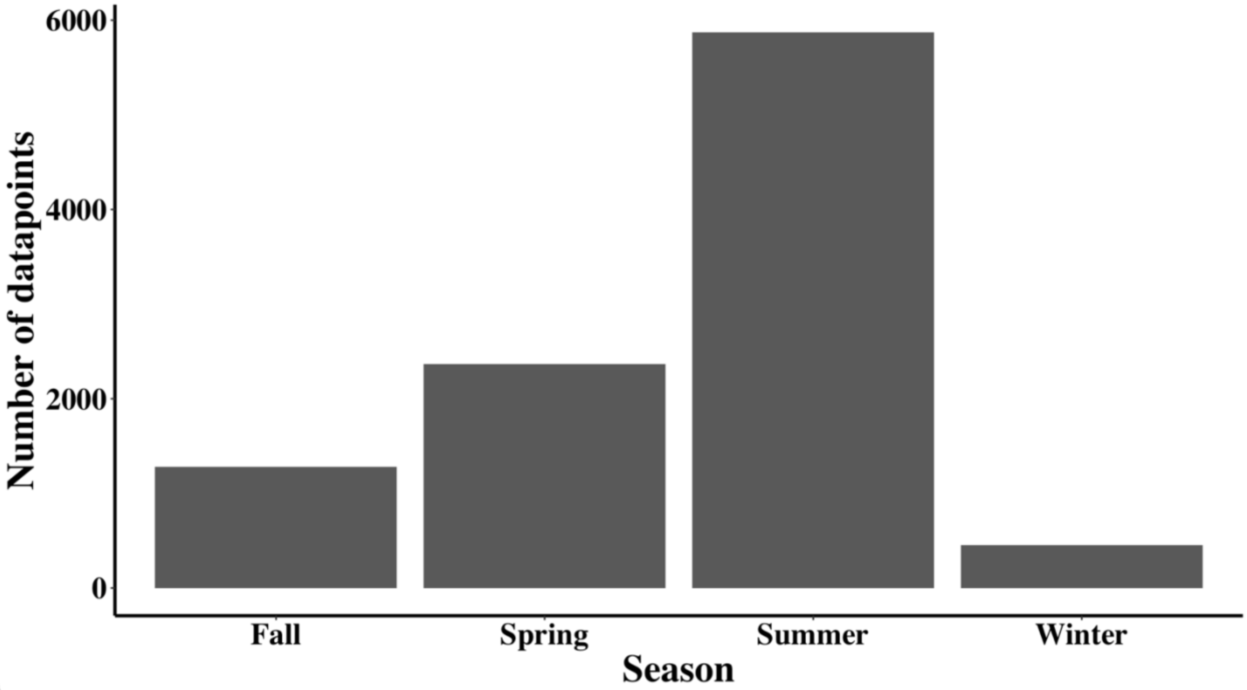

Fig. S2 Distribution of data points by season, based on collection date. Seasons are defined as Fall (September, October, November), Spring (March, April, May), Summer (June, July, August), and Winter (December, January, February). Each data point equates to an individual sample collected in a given year at a given location (latitude/longitude) and depth

Fig S3

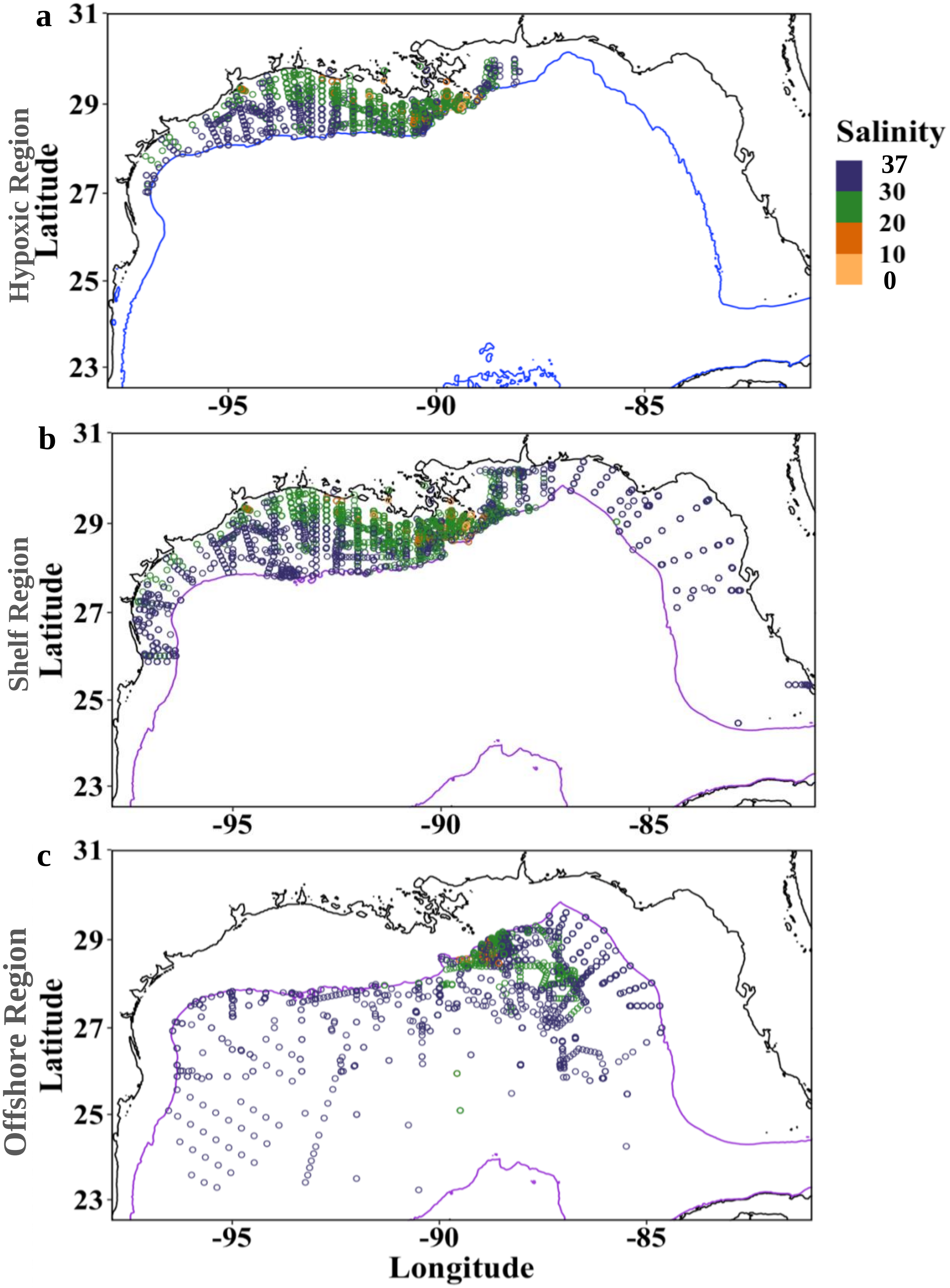
a
b
c
Hypoxic Region
Shelf Region
Offshore Region
Latitude
Longitude
31
29
27
25
23
-95
-90
-85
Salinity
37
30
20
10
0

Fig. S3 NGoM nutrient sampling stations 1985–2019 (excluding 1988 and 2014) for the (a) hypoxic, (b) shelf, and (c) offshore regions. Circles represent individual surface samples (0 - 5 meter collection depth). Circle color denotes sample salinity. Bathymetry isobaths denote the 60 meter (in a; blue line) and 200 meter (in b and c; purple line) depth isobaths. The hypoxic region is a subset of the shelf samples within longitudes -88° to -97° and latitudes 27° to 30° where bottom depths were < 60 meters (Rabalais & Turner, 2019; gulfhypoxia.net)

Figure S4

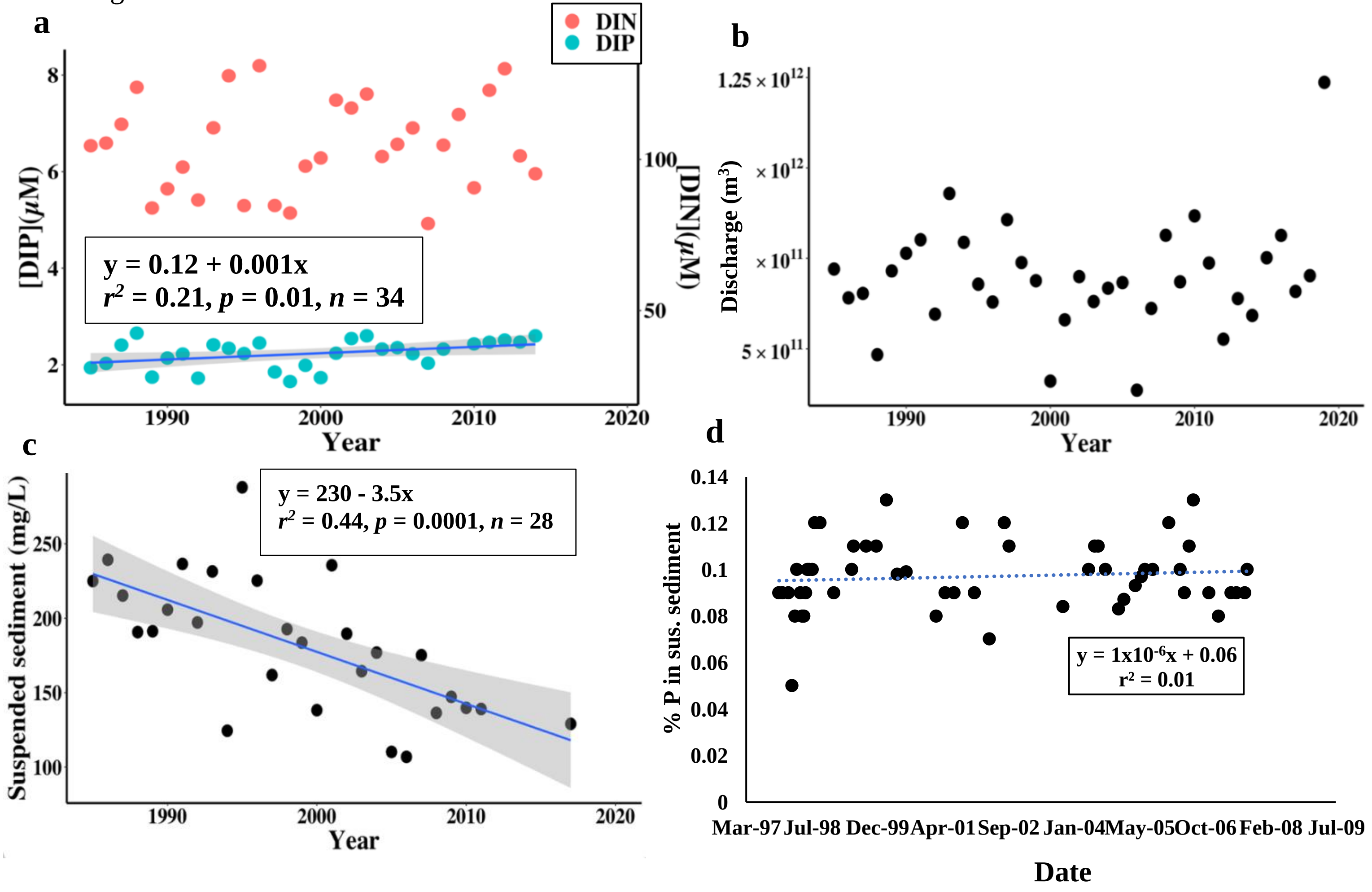

Fig. S4 Annual mean MAR (a) DIN (pink) and DIP (teal) concentrations at St. Francisville, LA, (b) discharge into the NGoM, (c) suspended sediment concentrations at St. Francisville, LA from 1985 to 2019 (see Figure 2 for annual mean nutrient loading into the NGoM 1985–2019), and (d) percentage P in MAR suspended sediment (digested) 1997 to 2009 . Gray shading represents the 95% confidence interval for the statistically significant regression lines. Non-significant regressions are not shown. Based on USGS data (Lee, 2022)

Figure S5

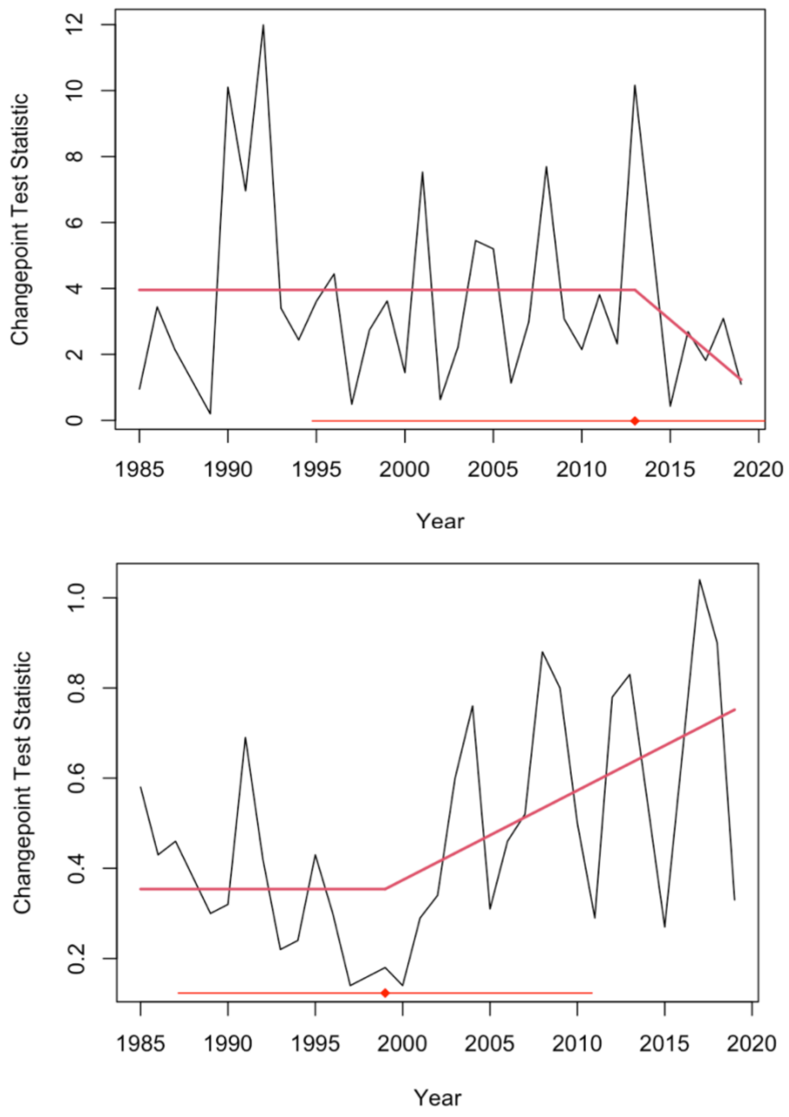

Fig. S5 Changepoint test statistics for NGoM (a.) annual mean DIN over time and (b.) annual mean DIP over time 1985–2019 (excluding 1988 and 2014); A linear regression model with segmented relationships were estimated using the *segmented()* R package for annual mean DIN over time and mean DIP over time to estimate a new regression model for broken-line relationships where the linear relationship changes. The DIN breakpoint is at 2013, and the DIP breakpoint is at 1999, though neither changepoint is significant. The red line above the x axis represents the error bars for the changepoint time statistics

Figure S6

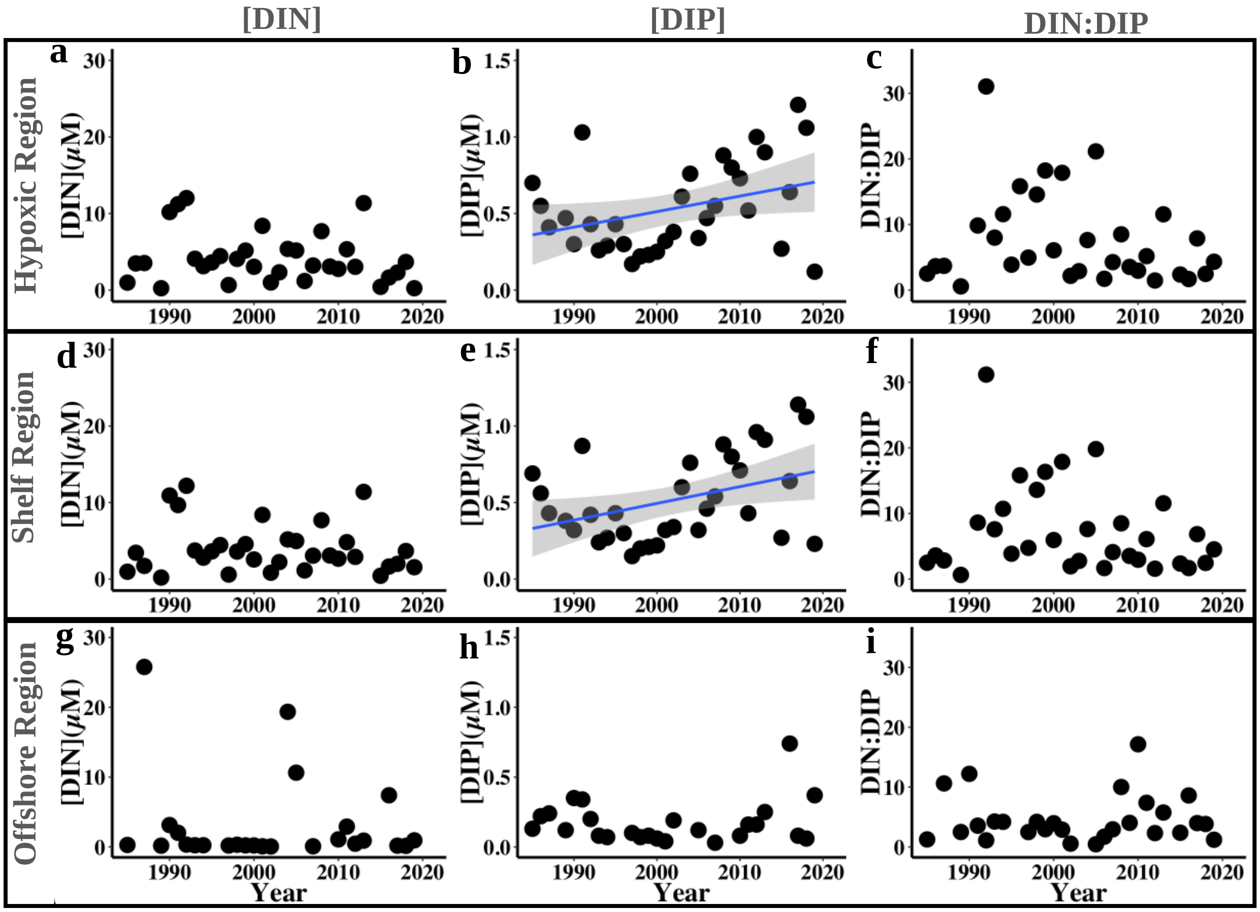

Fig. S6 Annual mean nutrient concentrations (DIN, DIP, and DIN:DIP) for each regional subset of the NGoM 1985–2019 (excluding 1988 and 2014); (a - c) hypoxic, (d - f) shelf, and (g - i) offshore region. Entire dataset annual mean nutrient concentrations (without regional subsets) are shown in Figure 3. Corresponding linear regression equations, r^2, *p*, and *n* values are listed in Table S2

Figure S7

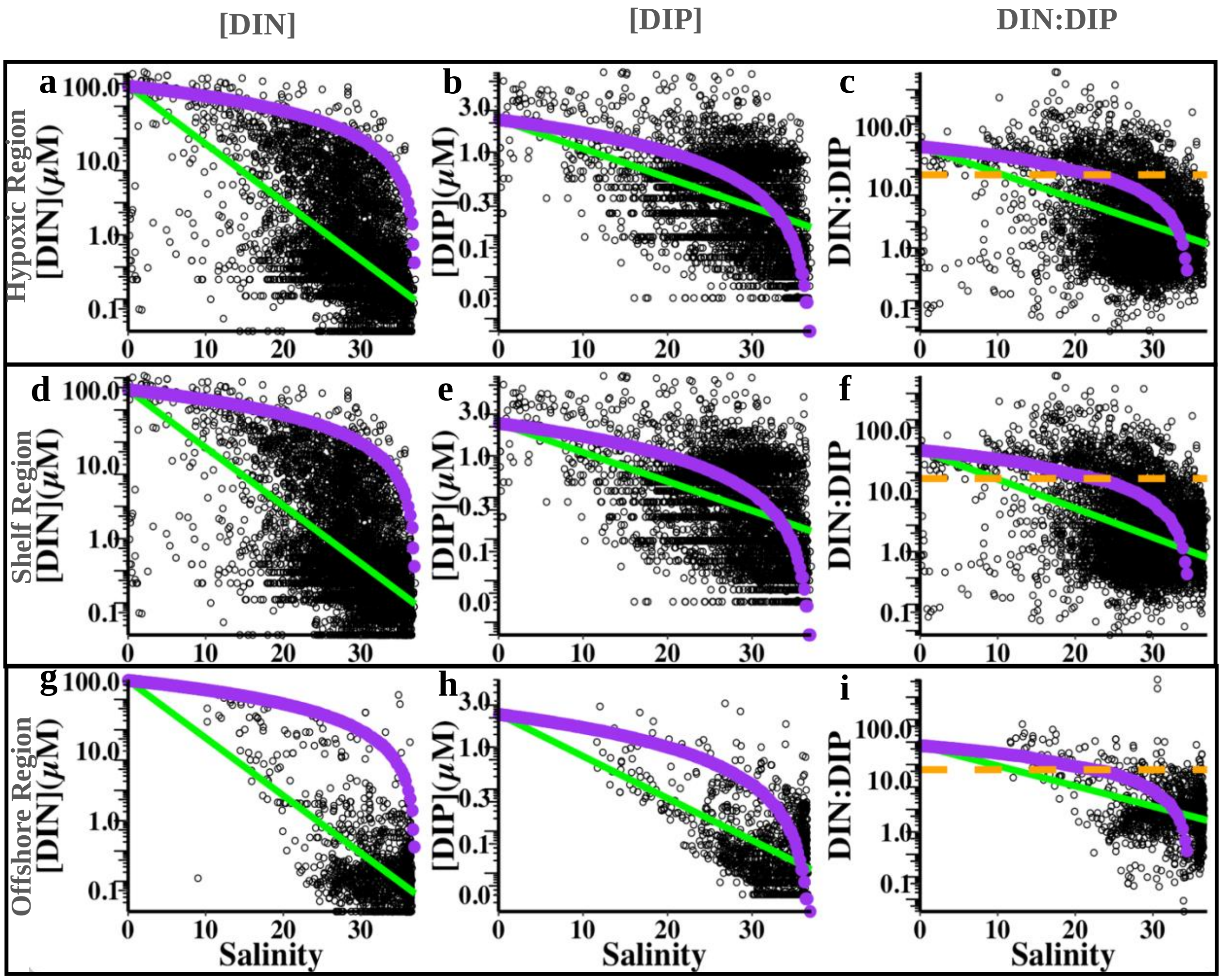

<table><tr><td>Fig. S7 NGoM surface nutrients vs. salinity by regional subset 1985–2019 (excluding 1988 and 2014): (a) hypoxic [DIN]; (b) hypoxic [DIP]; (c) hypoxic DIN:DIP; (d) shelf [DIN]; (e); shelf [DIP]; (f) shelf DIN:DIP; (g) offshore [DIN]; (h) offshore [DIP]; (i) offshore DIN:DIP – NGoM nutrient concentrations vs. salinity for all regions combined are shown in Figure 4. Black circles represent surface nutrient data, note the log scale of y-axes. The green lines represent linear regressions to ln-transformed data, with the corresponding equations, r^2, p, and n values listed in Table S7. The conservative mixing functions are shown in purple. In the DIN:DIP graphs (c, f, i), the orange dashed line represents the Redfield ratio of DIN:DIP = 16</td></tr></table>

Figure S8

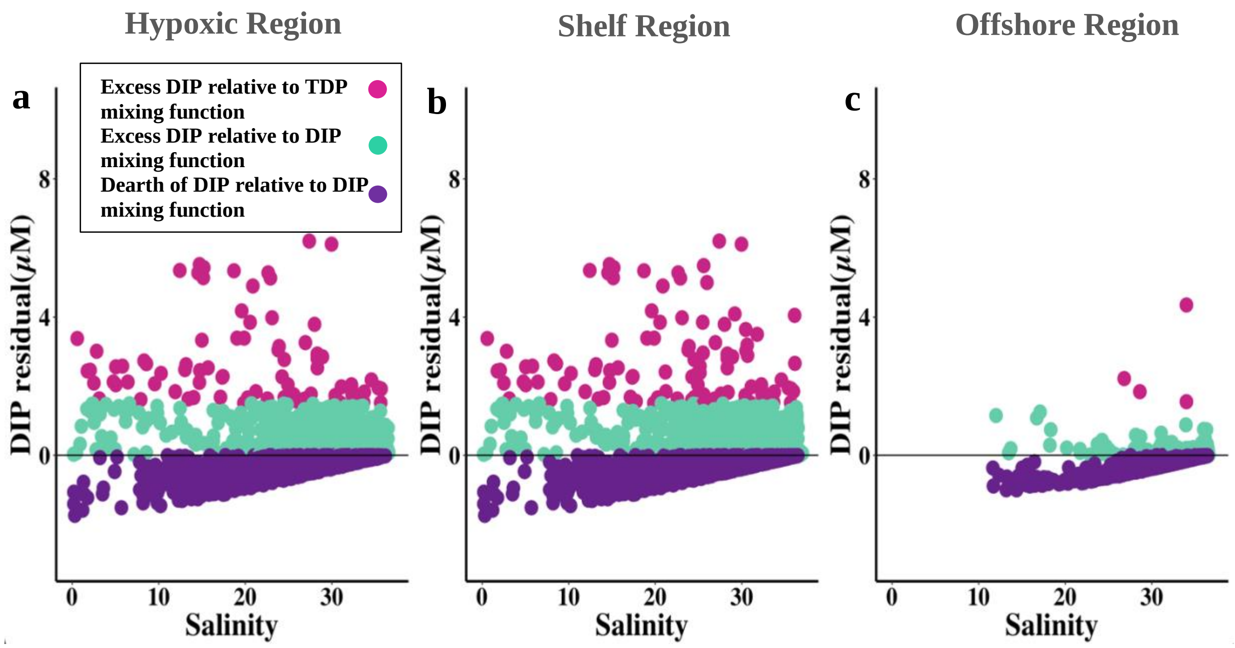

Fig. S8 Residuals of NGoM DIP concentrations (1985-2019) relative to the conservative mixing function connecting MAR DIP and TDP to the offshore endmember, data separated into (a) hypoxic; (b) shelf; and (c) offshore regions. Each point equates to an individual sample and shows the DIP residual relative to the MAR DIP mixing function (y = -0.059x + 2.2). Positive residuals (teal and pink) indicate that the actual values were higher than predicted, and negative residuals (purple) signify that the actual values were lower than predicted by the MAR DIP mixing function. Pink values are positive relative to both the MAR DIP mixing function and would also be positive relative to the MAR TDP mixing function (y = -0.089x + 3.3). Teal values are positive relative to the MAR DIP mixing function, but would be negative relative to the MAR TDP mixing function. Purple values are negative relative to both mixing functions

Figure S9

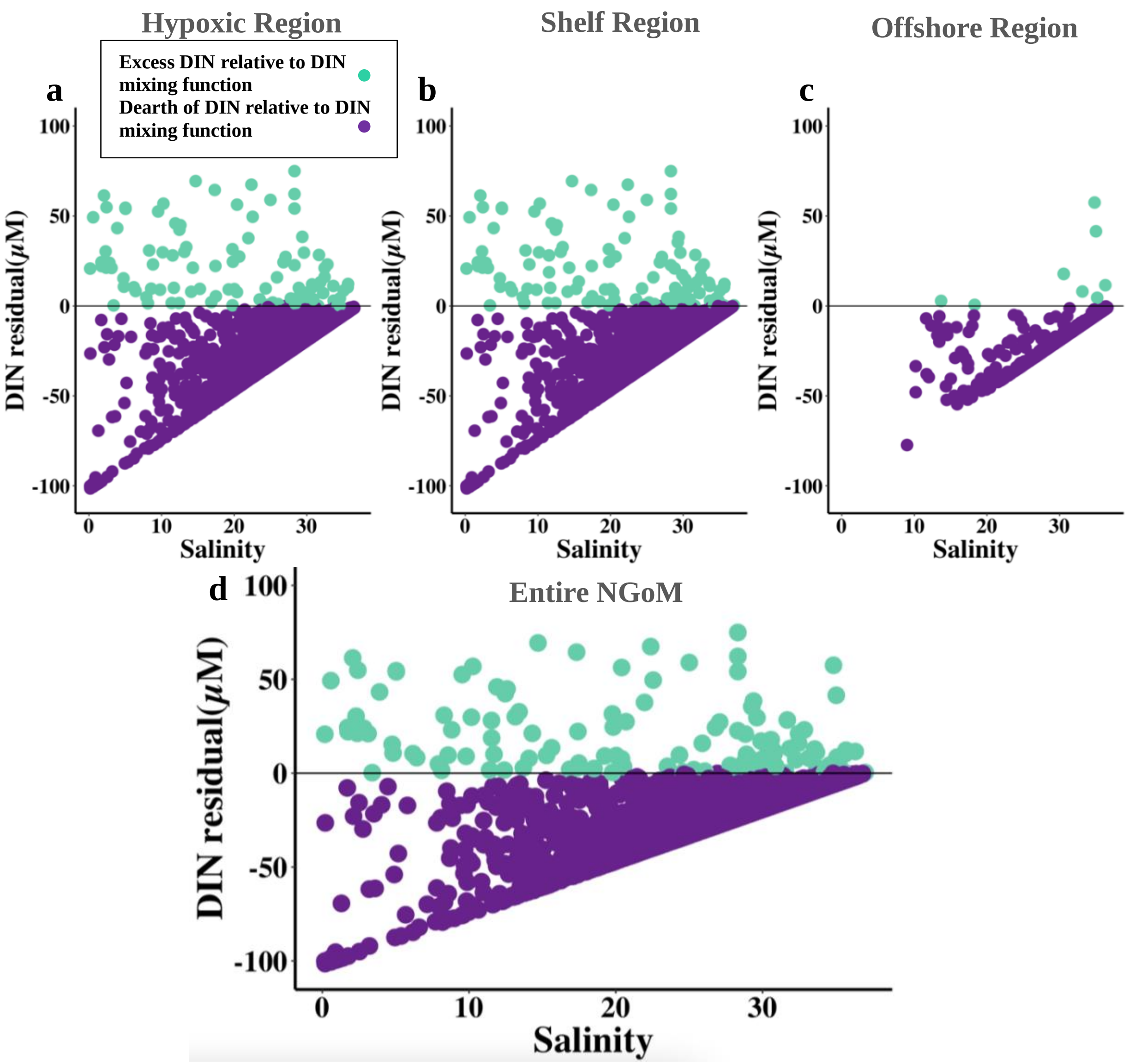

Fig. S9 Residuals of NGoM DIN concentrations (1985-2019) relative to conservative mixing function connecting MAR DIN to the offshore endmember, separated into (a) hypoxic; (b) shelf; (c) offshore regions; and (d) entire NGoM. Each point equates to an individual sample showing the residual relative to the MAR DIN mixing function ($y = -2.77x + 102.41$). Positive residuals (teal) indicate that actual values were higher than predicted, and negative residuals (purple) signify actual values were lower than predicted by the mixing function
